# Cancer disparities: Projection, COVID-19, and scenario-based diagnosis delay impact

**DOI:** 10.1371/journal.pone.0330752

**Published:** 2025-09-02

**Authors:** Ayse Arik, Andrew J. G. Cairns, George Streftaris

**Affiliations:** 1 School of Risk and Actuarial Studies, University of New South Wales, Sydney, New South Wales, Australia; 2 School of Mathematical and Computer Sciences, Heriot-Watt University, and Maxwell Institute for Mathematical Sciences, Edinburgh, United Kingdom; University of Auckland, NEW ZEALAND

## Abstract

There has been limited research on how disparities in cancer mortality may evolve in the future, although relevant socio-economic and regional disparities in cancer risk are well-documented. We studied future trends in breast cancer (BC) and lung cancer (LC) mortality up to 2036 across affluent and deprived communities in nine regions of England, motivated by the distinct socio-economic patterns and burden of these cancer types. We used cancer death registrations from the Office for National Statistics on population and deaths in nine regions of England by underlying cause of death from 2001 to 2018, stratified by sex, 5-year age group, and income deprivation. We applied a gender- and cause-specific Bayesian hierarchical model to obtain robust estimates of cancer mortality by age group, gender, deprivation quintile, and region, up to 2036. In these models, we also used a data-driven proxy for age-at-diagnosis as an additional risk factor, and non-smoker prevalence rates as a proxy for smoking.

We found that if pre-COVID conditions and trends remained the same, socio-economic disparities in LC would persist during our projection period. LC mortality rates for women in 2036 were found to be around 60% lower in the least deprived areas of London, as compared to the most deprived in the same region, with the disparities being even higher in northern regions and among men. Using data from the period 2011-2018, our model estimated 2% fewer LC deaths than those registered during the pandemic years (2020-2022) across England (and 4% fewer for men). Scenarios linked to delays in LC diagnosis led to stark differences in future excess mortality – significantly higher excesses in the northern regions compared to the southern regions, and in the most deprived areas compared to the least deprived areas. Additionally, our findings show that if pre-COVID conditions and trends remained unchanged, BC mortality would continue to decline up to 2036, with comparable rates in the regions of England. During the pandemic years, BC deaths were estimated to decline by 1% across England compared to the pre-pandemic trends (2001-2018). However, our analysis shows 10% to 13% increase in BC deaths for women aged 80+ in the same years.

Cancer disparities are predicted to persist in the future unless targeted interventions are implemented. Our results underscore the notable impact of delays in cancer diagnosis on cancer mortality and related inequalities. Future research that models different causes of death while adjusting model outputs for competing risk factors might be beneficial. Further models with individual-level socio-economic risk factors would also be useful.

## Introduction

Health inequalities, referring to unjust and avoidable differences in health across different population groups, has been one of the widely discussed topics across different fields. Without a solid answer having emerged for how to resolve widening health inequalities in different nations, the global COVID-19 pandemic started in Wuhan in December 2019 and rapidly spread to other parts of world in 2020 [1]. The pandemic has had a significant impact on vulnerable communities by potentially deepening long-lasting problems. Early empirical studies suggested that the COVID-19 pandemic has disproportionately affected certain groups, e.g. the elderly, people with comorbidities or those who are more deprived [2–7]. In the aftermath of the pandemic, official figures are pointing out statistically significant increases in the absolute gap in avoidable deaths between the most and least deprived areas compared to 2019, e.g. Fig 1 in [8].

**Fig 1:**
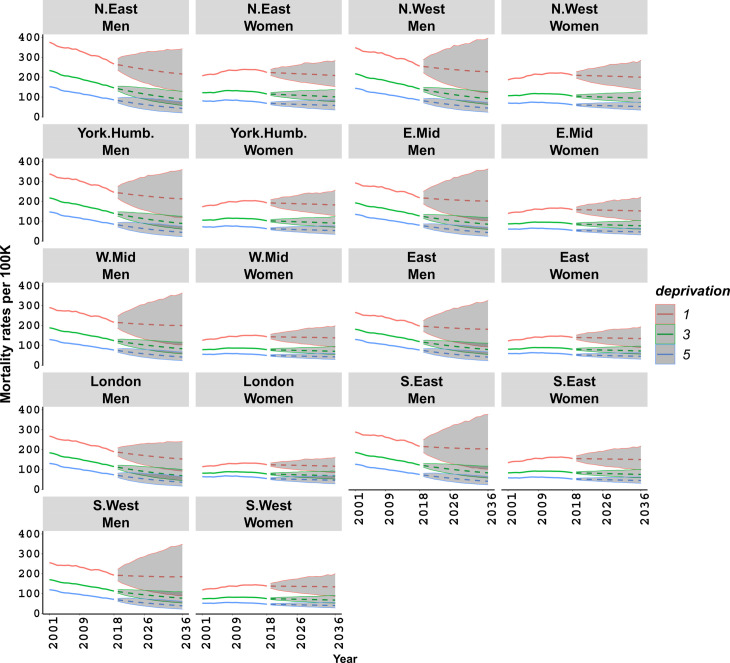
Age-standardised lung cancer mortality for men and women. Fitted (solid lines) and projected (dashed lines) rates, with 95% credible intervals, in selected deprivation quintiles 1 (most deprived), 3, and 5 (least deprived) and regions of England.

Cancer, apart from being one of the major causes of mortality and morbidity, representing 27–28% of all deaths in England per year, is also the largest driving cause of avoidable mortality [9,10]. The COVID-19 pandemic has affected cancer outcomes in a number of ways. The United Kingdom (UK) entered three national lockdowns as a response to the pandemic, and these measures were followed by changes in health practices leading to, for instance, a halt in cancer screening and considerable reductions in number of patients starting cancer treatment in the pandemic years [11]. Re-occurring national lockdowns and the changes in health services resulted in worrying patterns in cancer pathways, e.g. sharp declines in participants of cancer screening or fewer number of cancer patients starting a cancer treatment, in 2020 and 2021 [12]. For instance, urgent cancer referrals in the UK fell by up to 80% in 2020 [13]. In England, the number of new diagnoses between April and September 2020 dropped significantly compared to the same period in 2019 - by around 50% for breast cancer (BC) and approximately 30% for lung cancer (LC) [11]. These unprecedented changes sparked a fear of a shift to later cancer diagnosis. This has been a serious concern, since a late cancer diagnosis would restrict the opportunities for feasible treatment and worsen cancer survival.

Most of the recent published studies have focussed on identifying the impact of COVID-related health measures on cancer survival in England, based on National Health System (NHS) UK cancer registration and hospital administrative data. For example, [14] point out dramatic reductions in the demand for, and supply of, cancer services in response to the pandemic by showing that these reductions could largely contribute to excess mortality among cancer patients. [15] report a significant reduction in cancer survival as a result of treatment delay in England, whilst [13] note marked increases in avoidable cancer deaths as a result of diagnostic delays over a year on. [16] report significant increases in cause-specific cancer mortality as a result of diagnostic delays based on a population-based study in England. [17] further point out medium to large increases in BC mortality - ranging from 3% to 6% - among women aged 65 and above over a five-year period starting in 2020. These estimates are based on a modelling study calibrated using population data from England (2001–2019) and informed by relevant medical literature.

Another concern about disruptions in health services has been its broader implications on socio-economic inequalities in cancer risk, which has been a staggering issue in the last decades [16,18–24]. It has also been reported that the absolute gap in avoidable cancer mortality between the most and least deprived areas in England has widened in 2020 [8] due to, e.g., lower screening uptake in the most deprived areas [2].

In this study our focus is on understanding how socio-economic inequalities in cancer mortality are expected to change in future years. Given the major contribution of cancer risk to health disparities, a better understanding of these inequalities can provide insights into addressing broader health inequalities. Thus, we have developed detailed projection models for two major cancer types, with different characteristics, representing the largest percentage of overall cancer deaths in the UK: malignant neoplasm of trachea, bronchus, and lung, LC hereafter, and female malignant neoplasm of BC [25]. Specifically, we have two main interests: (a) providing a deeper insight into future LC and BC mortality based on a detailed population dataset; and (b) forecasting potential changes in future cancer disparities. Part of the contribution of this study is also providing a modelling framework in order to project LC and BC mortality on regional and deprivation level, where appropriate, under future scenarios that can be linked to delays in cancer diagnosis.

Our analysis is based on the population of England between 2001 and 2018, with data provided by the Office for National Statistics (ONS). We have developed cause- and gender-specific Bayesian hierarchical models to project cancer mortality, together with 95% credible intervals, where we use a Poisson distribution assumption for cancer deaths [16,26,27]. We investigated current and future patterns in cause-specific cancer mortality by year of death and various risk factors: age, gender, regions of England, income deprivation quintile, average age-at-diagnosis (AAD), and non-smoker (NS) prevalence rates. We also examined variations in cancer deaths during the COVID-19 years (2020–2022), along with the impact of diagnosis delays on cancer mortality under separate scenarios.

Importantly, socio-economic differences in LC mortality are predicted to persist in future years, whereas marginally significant regional differences would remain relevant for BC mortality. We have also shown the value of considering other risk factors, such as age-at-diagnosis, in the context of cancer mortality modelling by developing relevant scenarios in the projection models. Future scenarios are developed under LC modelling through the AAD variable, based on the assumption of delays in cancer diagnosis. As a result, excess LC deaths were estimated by age, gender, region, and deprivation levels of England, demonstrating marked variations across different population groups. Also, short-term changes in cancer deaths from 2020 to 2022 across England indicate a disproportionate impact on older age groups.

## Methods

This study uses anonymised, aggregate-level mortality and population data collected and provided by the UK (ONS) for the period between 4 July 2018 and 11 July 2023. We are third-party users of these data, which were accessed under a Data Access Agreement with the ONS. This agreement covers statistical data considered not to be identifiable microdata. The dataset does not contain individual-level or personally identifiable information. As such, informed consent was not required. Ethics approvals for the related research were granted by the Mathematical and Computer Sciences Ethics Committee at Heriot-Watt University under Project 53942 (‘Modelling, Measurement and Management of Longevity and Morbidity Risk’) and Project 8033 (‘Exploring Key Determinants of Mortality and Cancer Morbidity Disparities: Current Trends and Future Outlook’). At the time of data access and project approval, the use of anonymised, aggregate data provided by a national statistical authority did not require further ethical clearance.

### Population and mortality data

Data relating to this research were provided by the ONS in the UK during a period between 4 July 2018 and 11 July 2023, and were accessed for research throughout this period. The authors did not have access to information that could identify individual participants during or after data collection and subsequent analyses. We used cause-specific cancer death registrations where the underlying cause of death was recorded using the International Classification of Diseases (ICD-10), with codes C50 for breast cancer and C33-C34 for trachea, bronchus and lung cancer. Both death registrations and mid-year population estimates are available for each region in England, as described by the Nomenclature of Territorial Units for Statistics[28] specifically: north east, north west, Yorkshire and the Humber, East Midlands, West Midlands, east, London, south east, and south west. The data are stratified by five-year age-at-death group (20–24 to 85–89), single year from 2001 to 2018, gender, region, and income deprivation deciles (1 being the most deprived 10% of the population, to 10 being the least deprived 10%). We note that income deprivation has been found to be the most impactful domain on mortality, out of seven [29], in the national-level socio-economic index of multiple deprivation [30]. This data set is accessible up to 2022 at a lower granularity through an ONS service, namely ‘NOMIS’ [31].

Death rates for a given region and deprivation level were obtained by dividing cancer death registrations by the related mid-year population estimates. Death rates at the regional level were calculated by aggregating cancer death counts and relevant population estimates over deprivation levels.

### Smoking prevalence rates

Cigarette smoking remains the greatest cause of preventable death and disease globally, and is considered to be among significant risk factors for developing a number of diseases [32]. For instance, smoking is reported to be ‘the single biggest risk factor’ for, at least, 7 out of 10 LC cases [33,34]. Meanwhile, the association between BC risk and smoking is less strong, with higher risk depending on increasing amount of cigarette consumption, longer duration of smoking, and/or smoking initiation at young ages [35–37]. More evidence suggesting a potential causality between smoking and BC, especially in the case of a long-term heavy smoking starting at a young age, has been established in the past decade [38,39].

In this study we use smoking data by age and year, collected by the Health Survey of England[40], available for a longer number of years compared to the Annual Population Survey (APS), a large-scale continuous household survey in the UK. See Supporting information for further discussion on the APS-related smoking data. Importantly, we focus on NS prevalence rates as a proxy for smoking. This is to avoid modelling bias by: (a) maintaining consistent definitions of smoking information, and (b) ensuring a simple and clear interpretation of smoking in the implemented models [41,42].

We fitted a simple generalised linear model to account for the noise in the observed and fitted NS prevalence rates between 1981 and 2019. Observed and estimated age-specific rates aged between 45 and 75+ from 1981 to 2019 indicate an increasing trend in NS prevalence, with a more homogeneous and faster increase among men over the considered period (S2 Fig in S1 Text).

### Model structure

We have modelled cause-specific cancer deaths in men and women at different age groups under the Poisson distribution assumption [43]. For LC, the age groups are 45–54, 55–59,..., and 85–89, while for BC, the groups are 35–39, 40–44, ..., 85–89. This distinction is because the number of cancer causes differs by age, with significant mortality occurring from mid to older ages [44]. Differences in mortality across individuals born in the same year, and the heterogeneous structure in different sub-populations, are taken into account by implementing a Poisson-lognormal Bayesian hierarchical modelling structure[45–47]. Each Bayesian model is constructed by using main variables, namely age-at-death, year, deprivation quintile (1 being the most deprived 20% of the population, to 5 being the least deprived 20%), region, AAD, and NS prevalence rates, along with two-way interaction terms between selected main variables. While deprivation deciles could have been used, we opted for quintiles in order to ensure a larger number of observations for more robust modelling. The cause-specific cancer Bayesian models are determined by following a Bayesian variable selection procedure in the R-INLA software [48] based on two criteria: Deviance Information Criterion (DIC) [49], and Bayes factors [50]. The specified Bayesian modelling structures are fitted using Markov chain Monte Carlo methodology, implemented in the Bayesian analysis software WinBUGS [51].

Importantly, cause- and gender-specific cancer mortality rates are predicted beyond the observed period through a time series model featuring a random walk with drift for year-related effects [26], setting the baseline year as the last observed year (2018). For estimating future cancer mortality, we have assumed that region-, and deprivation-related effects would remain unchanged in the future.

Technical specifications of implemented models and full details of model assumptions can be found in Supporting information.

### Excess cancer deaths and scenarios under diagnosis delays

We have quantified short-term variations by age and region in both LC and BC deaths for the years 2020 to 2022 [52], based on a comparison between observed and expected cancer deaths under pre-pandemic trends.

We have also investigated the impact of cancer diagnosis delays on future cancer mortality in different socio-economic groups, motivated by the significant reductions in cancer registrations due to the initial health disruptions caused by the COVID-19 pandemic [53]. We have developed three scenarios associated with LC mortality by considering an increase in the AAD covariate (see S3 Fig for AAD in women and S4 Fig for men in Supporting information). This covariate is used as a proxy for age-at-diagnosis and implicitly captures incidence patterns. Detailed discussion on this variable can be found in the Supporting information. Specifically, we introduced a 1-month delay in AAD in the first scenario, a 3-month delay in the second scenario, and a 6-month delay in the third scenario. We have then estimated excess numbers of deaths, determined here as the difference between the expected numbers of deaths in a given scenario and those expected assuming pre-pandemic trends (as determined with our modelling between 2001 and 2018). We did not develop similar scenarios for BC, as the AAD variable showed no variability across regions of England, and therefore was excluded from BC modelling. Further details and technical definitions of excess deaths are provided in Supporting information.

## Results

We present rates projected up to 2036, using observed data from 2001 to 2018. Given the 18-year calibration period of observed data, and in line with best practice for maintaining forecast reliability and model validity, we have constrained the future projection accordingly. Our results initially focus on age- and gender-specific LC mortality rates split by deprivation quintiles and regions of England, and age-specific BC mortality rates by region. Age-standardised mortality rates presented here are based on the European Standard Population (ESP) 2013 as the reference population [54], for demonstrating mortality trends and inequalities across different deprivation levels and regions of England over time. Age-specific fitted and projected LC mortality rates across different deprivation quintiles and regions of England are provided in S7 Fig to S14 Fig for women and S17 Fig to S24 Fig for men, together with related Pearson residuals. Also, age-specific BC rates by region are available in S26 Fig to S32 Fig.

### Regional and deprivation level lung cancer mortality

Our results show a clear improvement in male LC mortality up to 2018, with reductions of about 25% and above 40% among the most and least deprived quintiles respectively, compared to 2001, across varying regions and deprivation levels. In contrast, no comparable improvement was observed for women. The rates in Fig 1 also show some recent mortality improvements for women, but only among the less deprived groups. Specifically, women in the least deprived quintiles saw reductions of around 15% across regions by 2018, with larger improvements in the southern regions of England. Women in the most deprived quintiles experienced a deterioration of around 10% in the same regions compared to 2001. Reflecting the clear declining trend in LC mortality among men in recent years, compared to largely levelled rates among women, the projected rates suggest greater future improvements for men, with varying degrees of progress across deprivation quintiles.

Importantly, our findings show that socio-economic differences persist in future years. This is evidenced by the estimated mortality rates in the most and least deprived quintiles over the projection period, which remain significantly different in each region.

### Regional level breast cancer mortality

**Fig 2:**
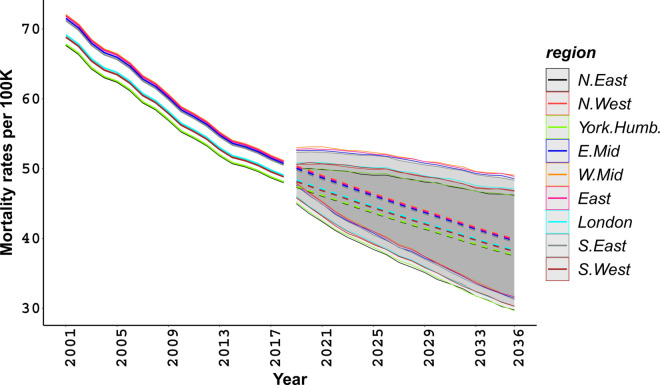
Age-standardised breast cancer mortality for women. Fitted (solid lines) and projected (dashed lines) rates, with 95% credible intervals, in regions of England.

Fig 2 presents age-standardised fitted and projected BC mortality rates from 2001 to 2036. The figure conveys two main messages. First, there is a declining trend in all regions over the past years, which is also expected to continue in the future. Second, region is a significant predictor for explaining differences in past BC mortality (see S3 Table in S1 Text), yet the differences across regions are marginal in the projection period, with wide uncertainty reflected in the overlapping credible intervals.

Despite comparable mortality predictions across regions in England, our estimates show persistent differences in the future for BC mortality among age groups [55], for example between the youngest screening age group (47) and the oldest age group (77) (S26 Fig in S1 Text).

### Disparities in cancer mortality

We have examined socio-economic differences in LC mortality over time. Varying regional and socio-economic disparities are evident; for example, a woman in the lowest income bracket in the north east of England is twice as likely to die from LC as compared to another women in a similar income bracket in London, with a widening absolute gap from 2001 to 2018 (Fig 1). To quantify these disparities both in past and future, we calculated relative differences in age-standardised mortality rates, ASMR, between the most deprived (quintile 1) and least deprived (quintile 5) groups as ASMR_*q*1_ − ASMR_*q*5_)/ASMR_*q*1_ for each gender and region.

**Fig 3:**
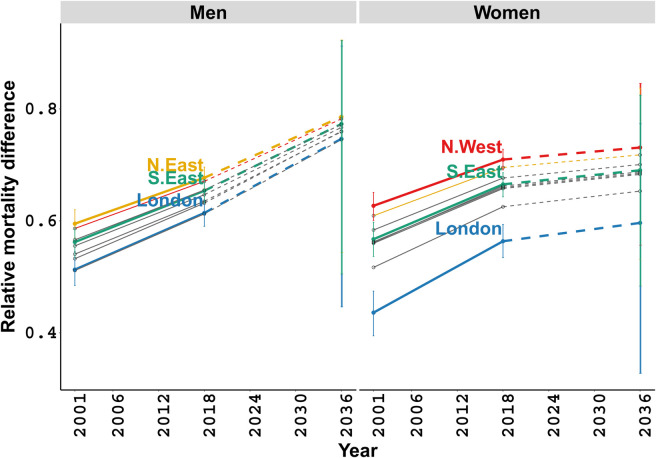
Relative differences in lung cancer mortality for men and women, between the most and least deprived quintiles. Fitted (solid lines) and projected (dashed lines) in 2001, 2018, and 2036 for the regions of England, with 95% credible intervals.

A wider relative deprivation gap in LC mortality among women is predicted as compared to their male counterparts throughout the inspected period. Both plots in Fig 3 display an increasing trend in the relative deprivation gap from 2001 to 2036 under the assumption of unchanged pre-pandemic conditions. However, this trend seems to slow down for women after 2018 compared to the earlier estimates. Notably, in the last observed year, there is a significant discrepancy in the relative deprivation gap for women across the north west, the south east of England, and London, while other regions show comparable estimates. Meanwhile, for men, substantial differences are estimated between the north east of England and London, with similar outputs in other regions, in the same year. Last, our projection results point out comparable deprivation gap for both genders across different regions by 2036.

### Excess deaths

#### Short-term variations in cancer deaths during the COVID-19 years.

We have assessed the short-term variation in number of LC and BC deaths during the period 2020–2022 by comparing observed annual death counts to model-based expected deaths, where the model is fitted to data from 2001 to 2018. We note that our underlying data are annual, and comparisons are made using officially registered, publicly available annual death records. In particular, we have calculated the ratio of observed numbers of deaths, divided by the corresponding expected numbers, by region and age group.

We found a 2% decrease in LC deaths among women and a 4% decrease among men across all regions in England during the three pandemic years (2020–2022), compared to the expected deaths based on the LC patterns between 2001 and 2018 (S4 Table and S6 Table in S1 Text). The corresponding ratios are statistically close to 1, indicating a tentative decrease, likely due to the fact that ‘a larger number of people died than usual (due to the Covid-19 pandemic)...’ in 2020 and 2021 (p. 12 in [56]). However, there are important variations. For instance, LC death rates for women were 3–6% higher than expected in the East Midlands, West Midlands, and the south west of England. In contrast, significant declines were observed in London (16% for women, 11% for men) and in the north east of England (8% for women) (see S4 Table for further results). We also found marginal increases in LC deaths for women aged 70–74, 80–84, and 85–89, changing between 2 to 7% with a lower degree at older age groups (S5 Table and S7 Table). Important reductions have occurred between ages 55 and 64 for both genders.

We estimated a slight decrease (1%) in BC deaths across England during 2020–2022 (S8 Table and S9 Table). A closer examination reveals that three regions (the north east of England, Yorkshire and the Humber, and the East Midlands), have witnessed a marginal increase in BC deaths (changing between 1–5%). Last, Table 1 shows that older women (80–89) have been disproportionately affected with notable increase in BC deaths (10–13%) from 2020 to 2022, whereas younger women, such as those aged 40–44, saw a significant decline (9%). Regional and age-specific short-term variations in BC deaths, along with 90% credible intervals, are detailed in S9 Table and S10 Table, respectively.

**Table 1:** Short-term variations in breast cancer deaths at different ages in England from 2020 to 2022, with 95% credible intervals.

	Registered deaths	Expected deaths	Mean ratio: registered/expected
Ages 35–39	391	395.05	0.99 (0.91, 1.08)
Ages 40–44	642	703.82	0.91 (0.84, 0.99)
Ages 45–49	1156	1094.09	1.06 (0.98, 1.15)
Ages 50–54	1755	1759.06	1.00 (0.92, 1.08)
Ages 55–59	2178	2427.11	0.90 (0.84, 0.97)
Ages 60–64	2192	2542.24	0.86 (0.80, 0.93)
Ages 65–69	2310	2547.13	0.91 (0.84, 0.98)
Ages 70–74	3099	3222.41	0.96 (0.89, 1.04)
Ages 75–79	3475	3533.54	0.98 (0.91, 1.06)
Ages 80–84	3673	3347.56	1.10 (1.02, 1.18)
Ages 85–89	3459	3068.50	1.13 (1.05, 1.22)

#### Impact of diagnosis delays: Lung cancer.

We investigated excess LC deaths in men and women due to diagnosis delays of 1 to 6 months, potentially resulting from significant health service disruptions (e.g. [57]). Such delay duration is consistent with the literature (e.g., [58–61]). Excess deaths are defined as the difference between expected deaths under delayed-diagnosis scenarios and those based on pre-pandemic trends.

Our modelling predicts 2,340 (1,743; 2,869) and 10,180 (7,944; 12,340) cumulative excess LC deaths for women in England due to 1-month and 6-month diagnosis delays, respectively, over 17 years, from 2020 until 2036. In the same period, a 1-month delay in diagnosis is estimated to result in 5,164 (4,353 to 6,066) cumulative excess LC deaths for men, with 28,660 (23,040 to 35,090) excess deaths with a 6-month delay in diagnosis.

**Fig 4:**
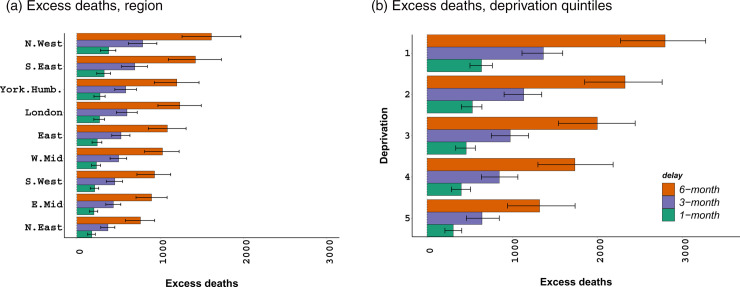
Cumulative lung cancer excess deaths for women from 2020 to 2036. Total excess deaths (over 17 years) in different deprivation quintiles and regions of England, with 95% credible intervals.

Cumulative excess deaths for women are plotted for the regions and deprivation quintiles in England in Fig 4. The left-hand plot in Fig 4 shows considerable differences in excess deaths (a) as a result of 1- to 6-month diagnosis delays in a given region; and (b) by region, such as the south east vs. north east of England. The right-hand plot additionally shows marked differences between the most and least deprived quintiles as a result of any delay in diagnosis. Our model has estimated higher numbers of excess deaths for men, with comparable conclusions (S33 Fig in S1 Text).

**Fig 5:**
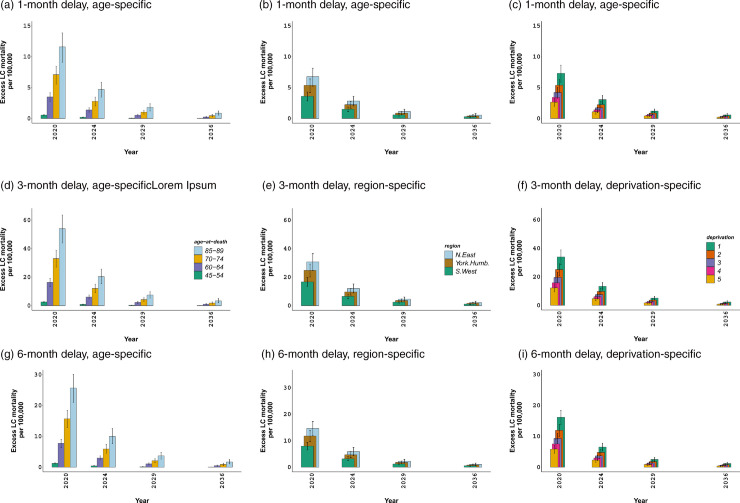
Lung cancer excess mortality, per 100,000 women, by age-at-death, selected regions and deprivation quintiles in England. Annual excess deaths from 2020 to 2036, with 95% credible intervals. Note that differences in lung cancer excess mortality at other ages, in other regions or deprivation quintiles in intermediate years, are comparable to the presented years.

We also show LC excess mortality, per 100,000 women, by age, region, and deprivation quintiles in single projection years (Fig 5). The first row of the figure shows the relevant results for a 1-month diagnosis delay, while the second and third rows demonstrate the results associated with a 3-month and a 6-month diagnosis delay respectively. Although excess mortality rates for men are significantly higher than those for women, the distribution of these rates by age, region, and deprivation is comparable to the female counterparts (see S34 Fig in S1 Text).

Fig 5a and 5g point out markedly different excess mortality, per 100,000 women, across the middle, e.g. 45–54, and old age groups, e.g. 70–74. Fig 5b and 5h show the lowest excess mortality in the south west of England in 2020, with significantly higher excess mortality in the north east. Finally, Fig 5c and 5i point out substantial differences in excess deaths, up to a factor of 4, across the most and least deprived quintiles as a result of diagnosis delays.

## Discussion

This paper has focussed on two primary cancer types: LC and BC mortality. We have utilised population data in England at regional and deprivation levels, between 2001 and 2018. Accordingly, we have carried out a detailed analysis to understand how inequalities in cancer mortality are expected to change in future. We have quantified short-term variations in cancer deaths by age and region between 2020 and 2022. Additionally, we have explored scenarios concerning LC modelling that pertain to delays in cancer diagnosis, aiming to assess the impact of such delays on LC mortality up to 2036.

### Main findings

We found evidence suggesting that socio-economic differences in LC mortality for both genders have increased, with notable differences across the regions of England in the years until 2018, and are expected to continue having similar increasing trends up to 2036 (Fig 3). Meanwhile, regional variation in BC mortality is predicted to remain relevant up to 2036. Furthermore, we estimated a marginal decrease in LC and BC deaths during 2020–2022, compared to the pre-pandemic trends from 2001 to 2018. However, notable variations were predicted across different population groups, particularly by region, such as London, and among older age groups (aged 70 and above). We also found that delays in cancer diagnosis would lead to significantly higher excess deaths from LC, varying widely for delays of 1 to 6 months. Importantly, the related future-projected LC excess deaths were found to be substantially

- higher at older age groups including 60–64 years old;
- higher in the northern regions of England compared to the southern regions; and
- higher for those living in the most deprived quintiles compared to those in the least deprived quintiles.

### Strengths and limitations of this study

We employed a Bayesian hierarchical modelling approach, stratified by age, year, gender, region, and deprivation level, enabling us to provide a comprehensive analysis that goes beyond existing empirical evidence. This framework is robust in its capacity to analyse nuanced data patterns, making it ideal for examining intricate factors like regional and socio-economic disparities in cancer mortality.

Our model is built on general population data, and it allows us to address parameter uncertainty and incorporate multiple predictive variables, including complex interactions to capture non-linear relationships. For instance, NHS Digital recently revealed stark disparities in age-standardised cancer mortality in England, with the highest rates observed in the most deprived areas in 2020, showing a widening deprivation gap compared to 2019 [62]. Also, LC was reported to be particularly impacted, showing consistently higher mortality in deprived areas. Aligned with these patterns, our estimates of LC deaths show the highest mortality in the most deprived quintiles with persistent deprivation gap in future. Additionally, our estimates further offer a detailed comparison for both men and women at various age groups living in different regions of England, together with 95% credible intervals.

Our approach provides a comprehensive analysis for both LC and BC mortality, along with estimates of the potential impact of diagnosis delays on LC mortality across demographic, socio-economic and regional levels, using scenario-based modelling. In the absence of detailed data on cancer referrals or diagnostic timelines, we used the AAD variable as a proxy for LC diagnosis. While AAD is statistically significant and informative at the population level, it captures average effects and does not represent observed individual-level diagnostic delays. This limitation should be considered when interpreting our pandemic-related scenarios. Nonetheless, our scenario-based approach offers valuable insights into the potential impact of late diagnosis on LC mortality, under the implicit assumption of no cancer treatment. It highlights sharp increases in projected deaths, consistent with recent findings in other countries (e.g., [63–65]).

We also quantified short-term changes in LC and BC mortality between 2020 and 2022, finding varying outcomes on age- and region-specific levels. These findings would offer valuable insights for public health planning in future years.

We note challenges in accessing further cancer data at both deprivation and regional levels in the most recent years. Also, smoking data have not been available in the same granularity as for cancer data. This affected our ability to fully account for variations in smoking behaviour, demographic shifts, and the latency between exposure and disease onset. While our approach - using lagged non-smoker prevalence - is consistent with practice in the literature and provides a reasonable approximation of the smoking-related impact on cancer mortality, the estimates may still involve some degree of over- or under-estimation in specific population groups when considered in isolation. Similar challenges appeared when mid-year population estimates were required by deprivation quintiles for obtaining excess deaths. Nonetheless, suitable adjustments have been made by following similar practice in the available literature [66]. Although the population exposure in our projections for 2019–2036 account for certain demographic changes - such as population growth in most areas (particularly in the south and Midlands) and among older age groups (65 and over) [67] - these estimates may be conservative. In particular, the ONS has reported continued population growth in England and Wales in recent post-pandemic years, primarily driven by net international migration [68]. These changes may alter the socio-economic composition of population structure, potentially influencing future projections of cancer mortality. For example, while neighbourhood-level income deprivation was not significant in explaining changes in BC mortality, individual-level income variables could prove important. Additionally, we have not considered competing risk factors in our modelling approach.

There are several important areas for further research. First, our study does not include individual-level socio-economic variables, such as household income. Incorporating individual-level risk factors could enhance the predictive power of established models and might lead to different modelling structures. Additionally, addressing the decline in cancer deaths due to a new cause, such as COVID-19, and examining how future trajectories could evolve as a result, would significantly broaden the scope of this research.

## Conclusion

Modelling and identifying future trends in cancer mortality is important for outlining expected socio-economic and regional variations in cancer risk in the future. Our findings can contribute towards informing policy making for targeted health initiatives that can implement evidence-based, cancer-specific interventions to efficiently address broader health inequalities. This is closely linked to the objectives set by the Levelling Up White Paper, which aims to improve healthy life expectancy in the UK by 5 years by 2035 [69].

Our results can also help towards better understanding and assessment of the implications of late diagnosis for cancer mortality and survival rates. This is relevant to long-term health care planning, which has been a particular concern since the unprecedented health disruptions in the earlier years of COVID-19. Investigating differences in excess cancer mortality among the most and least deprived population groups can provide valuable insights into possible future gaps in mortality between the poorest and most affluent population groups.

## Supporting information

S1 Text Methodology and further results. (PDF)
