# Supplementary material for: Cancer disparities: Projection, COVID-19, and scenario-based diagnosis delay impact
Source: PLoS One. 2025 Sep 2;20(9):e0330752. doi: 10.1371/journal.pone.0330752 (PMC12404486; doi:10.1371/journal.pone.0330752)
Supplement: S1 Text — (PDF) [file pone.0330752.s001.pdf]

Supporting information: methodology and further results

Cancer disparities: projection, COVID-19, and scenario-based diagnosis delay impact

Ayşe Arık, Andrew J.G. Cairns, George Streftaris

A simple model for smoking prevalence rates

Smoking differs by deprivation levels [1] in England, along with age and gender, over time. According to the Annual Population Survey (APS) [2], smoking prevalence has declined in each deprivation decile since 2012, with statistically higher smoking prevalence in the most deprived neighbourhoods as compared to the least deprived ones in England (Fig S1).

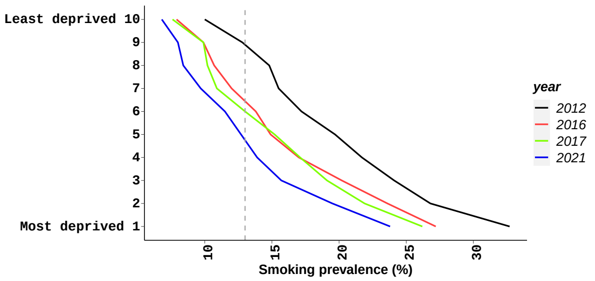

S1 Fig. Prevalence rates of current smokers by deprivation deciles. All persons aged 18 years and over, England, 2012 to 2021, where the dashed line is the national average, 13%, in 2021. Source: Annual Population Survey from the Office for National Statistics.

In the absence of comprehensive data, we use publicly available data by age and year provided by the Health Survey of England [3]. Specifically, we use non-smoker (NS) prevalence rates as a proxy for smoking in the implemented projection models. Also, we assume a lag of 20 years while accounting for contribution of NS prevalence to cancer mortality risk [4]. This means, for example, NS prevalence for men in 1981 must be used as an input to estimate, e.g., male LC mortality in 2001. However, the available data from the Health Survey of England starts from 1993 [3]. Provided we don't have access to the data before 1993, we relied on a simple modelling approach. Particularly, first, we have applied a gender-specific linear model to the available dataset between 1993 and 2019 as

$$\mathrm{NS}_{a,t} = \beta_0 + \beta_{1,a} + \beta_2 t + \beta_3 t^2 + \beta_{4,a} t, \tag{1}$$

and then the parameter estimates in (1) are employed to back estimate NS prevalence to 1981 for each gender.

Fig S2 presents the observed and estimated age-specific rates for men and women aged between 45 and 75+ during the period 1993–2019, along with back-estimates for 1981–1993.

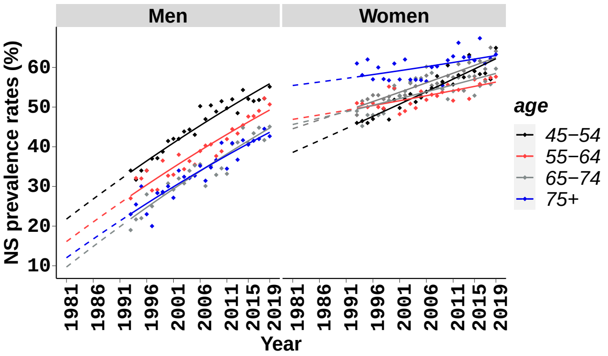

S2 Fig. Non-smoker prevalence rates at selected age groups between 1981 and 2019. Observed rates (dots), fitted rates (solid lines), and back-estimated rates (dashed lines).

Average age-at-diagnosis

Age at diagnosis is a well-established determinant of cancer survival. In [5], a link between cancer morbidity and mortality was established using a variable termed average age-at-diagnosis (AAD). Work in [5] demonstrated that AAD is a statistically significant predictor of type-specific cancer mortality. In the present study, AAD is incorporated into our projection models as a covariate serving as a proxy for age-at-diagnosis. Its inclusion was supported by formal variable selection procedures, confirming its relevance in explaining variation in the current dataset.

AAD for gender g in deprivation quintile d of region r at the time of diagnosis t, denoted by $\text{AAD}^{\text{morbidity}}_{g,d,r,t}$, is estimated as follows:

$$\text{AAD}^{\text{morbidity}}_{g,d,r,t} = \frac{\sum_a a\hat{\lambda}_{a,g,d,r,t} E^{\text{std}}_a}{\sum_a \hat{\lambda}_{a,g,d,r,t} E^{\text{std}}_a}, \tag{2}$$

where E^{std}_a shows population numbers at age-at-diagnosis a according to the ESP 2013, and $\hat{\lambda}_{a,g,d,r,t}$ is the relevant type-specific fitted incidence rate obtained based on the best fitted models in [5]. For modelling purposes, AAD is then weighted over years as described below:

$$\text{AAD}^{\text{morbidity}}_{g,d,r} = \frac{\sum_t \text{AAD}^{\text{morbidity}}_{g,d,r,t} E_{g,d,r,t}}{\sum_t E_{g,d,r,t}}, \tag{3}$$

by using the relevant mid-year population estimates $E_{g,d,r,t}$ in deprivation quintile d of region r. Note that, if deprivation is not a significant variable in the model under inspection, AAD could also be averaged over deprivation quintiles so that it would be by region only.

In this study, the AAD variable is found to be significant in explaining differences in LC mortality but not in BC mortality. Fig S3 shows estimated AAD values in LC for women from 2001 to 2017. We note that 2017 is the latest available calendar year in the LC morbidity data. We estimate an increasing trend in AAD values over the calendar years, with comparable results in the regions of England. However, the estimates across deprivation quintiles in a given region are notably different, where lower AAD values are calculated in more deprived quintiles.

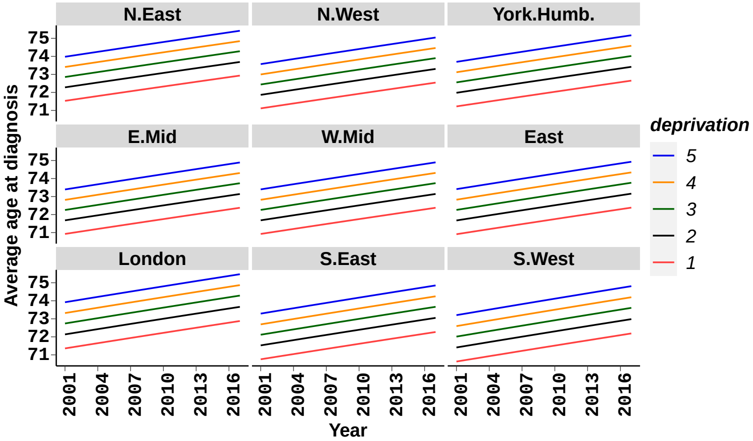

S3 Fig. Average age-at-diagnosis in lung cancer mortality, females, in deprivation quintiles 1 (most deprived) to 5 (least deprived), of regions of England between 2001 and 2017.

Fig S4 demonstrates AAD estimates in LC for men between 2001 and 2017. Similar to the female counterparts, there is an increasing trend in calculated AAD values over the time, with lower AAD values estimated in more deprived quintiles of a given region. We note higher AAD estimates for men as opposed to women.

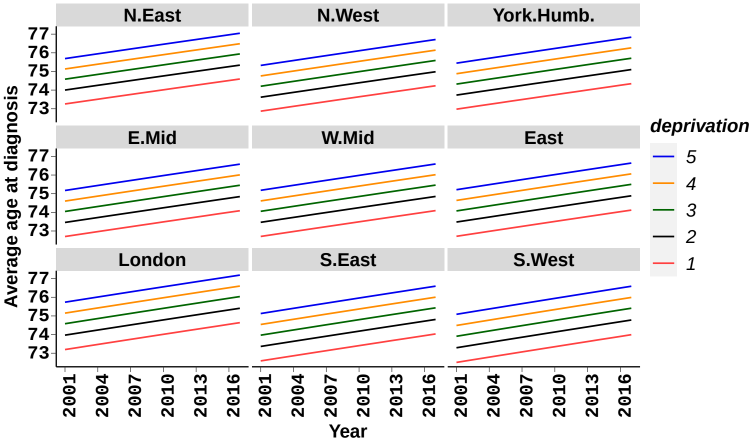

S4 Fig. Average age-at-diagnosis in lung cancer mortality, males, in deprivation quintiles 1 (most deprived) to 5 (least deprived), of regions of England between 2001 and 2017.

Projection models for cancer mortality

We assume that the number of gender- and cause-specific cancer deaths $D_{a,d,r,t}$ at age a and year t in deprivation quintile d of region r in England follows a Poisson distribution. Although this is a common assumption in the literature since the study of [6], there is an issue with this assumption. Specifically, the underlying assumption imposes mean-variance equality such that

$$\hat{\mathbb{E}}(D_{a,d,r,t}) = \hat{\text{var}}(D_{a,d,r,t}) = \hat{\theta}_{a,d,r,t}\ P_{a,d,r,t},$$

where $\hat{\theta}_{a,d,r,t}$ shows the expected mean of gender- and cause-specific fitted mortality rates and $P_{a,d,r,t}$ shows the related mid-year population estimates. This suggests that individuals born in the same year could have the same mortality experience despite several different factors, such as smoking, income, and education, impacting mortality [7–9]. This leads to an additional variation across individuals, also known to be 'overdispersion'.

In order to deal with overdispersion, we have constructed a baseline model for a given gender and cause-specific cancer mortality using a Poisson-lognormal Bayesian hierarchical model. The general structure of our model is:

$$\begin{aligned} D_{a,d,r,t} &\sim \text{Poisson}(\theta_{a,d,r,t}\ P_{a,d,r,t}) \\ \theta_{a,d,r,t} &\sim \text{Lognormal}(\mu_{a,d,r,t}, \sigma^2) \\ \mu_{a,d,r,t} &= \boldsymbol{\beta X} \\ \sigma^2 &\sim \text{Inv.Gamma}(1, 0.1) \\ \boldsymbol{\beta} &\sim \text{Normal}(0, 10^4), \end{aligned} \tag{4}$$

where

- $\theta_{a,d,r,t}$ is the gender-specific mortality rates at age-at-death a in year t in deprivation quintile d of region r, whenever applicable;
- $\mu_{a,d,r,t}$ is the location parameter of lognormal distribution for a given cancer type, which is defined based on different covariates, denoted by $\boldsymbol{X}$, and associated model parameters $\boldsymbol{\beta}$, except those linked to the period effect, t, which are separately denoted as $\boldsymbol{\kappa}$;
- non-informative prior distributions are assumed for model parameters $\boldsymbol{\beta}$ and σ^2, where appropriate, to reflect relative prior ignorance on their values.

The structure of the location parameter, $\mu_{a,d,r,t}$, differs for each cause-specific cancer with respect to related main variables, i.e. age-at-death, year, deprivation quintile, region, AAD, and NS prevalence rates, and relevant two-way interaction terms between them. For example, $\mu_{a,d,r,t}$ might have the following form:

$$\mu_{a,d,r,t} = \beta_0 + \beta_{1,a} + \beta_{2,r} + \beta_{3,d} + \beta_4 \text{AAD}_{r,d} + \beta_5 \text{NS}_{a,t-20} + \kappa_t + \text{interaction terms}, \tag{5}$$

where age-at-death, region, and deprivation quintile are considered as categorical variables, and NS prevalence rates and AAD variable are assumed to be numerical variables, standardised to have zero mean and unit variance to facilitate the calculations. The period effect, k_t, allows for projection into future years and is determined using a random walk with drift, as described in Eq 6. Full specifications of parameter μ for LC and BC mortality are provided between Eq 8 to Eq 10.

We note that AAD is a model-driven metric obtained from related population cancer morbidity to represent age-at-diagnosis in the absence of comprehensive data for cancer mortality modelling. This variable is introduced and explained in [5]. Also, for model identifiability and interpretability, sum-to-zero (STZ) constraints are imposed to all categorical variables, apart from the period effect. The STZ constraints enable comparisons between a given level of a categorical variable and the corresponding average effect as the reference level.

In our model, we describe the period effect in (5) using a random walk with drift. Depending on the model specification, there can be more than one coefficient related to

period effects, denoted by $\boldsymbol{\kappa} = (\kappa_{1,t}, \kappa_{2,t})^T$, between 2001 and 2018. Specifically, we assume that each period effect, $\kappa_{i,t}$ for $i = 1$, 2, follows the process below:

$$\begin{aligned} \kappa_{i,t} &= \psi_{\kappa_i} + \kappa_{i,t-1} + \epsilon_{\kappa_i,t} \\ \psi_{\kappa_i} &\sim \text{Normal}(0, {\sigma^2}_{\psi_{\kappa_i}}) \\ \epsilon_{\kappa_i,t} &\sim \text{Normal}(0, \sigma^2_{\kappa_i}) \\ \sigma^2_{\kappa_i} &\sim \text{Inv.Gamma}(1, 0.001). \end{aligned} \tag{6}$$

Here, the variance of the drift term is estimated as $\hat{\sigma}^2_{\psi_{\kappa_i}} = \frac{1}{2018-2001}\,\hat{\sigma}^2_{\kappa_i}$. We adopt corner constraint for the period effect $\boldsymbol{\kappa}$ such that $\boldsymbol{\kappa}_1 = (\kappa_{1,0}, \kappa_{2,0})^T = (0,0)^T$.

Under the assumption of unchanged age-at-death-, deprivation- and region-level patterns in cancer risk, the future mortality rates for a given gender and cancer type can be derived as

$$\theta^*_{a,d,r,t} \sim \text{Lognormal}(\mu^*_{a,d,r,t}, \sigma^2),$$

where a new location parameter, $\mu^*_{a,d,r,t}$, is defined considering changes only in time-related terms. To be precise, the period-related effects would be extrapolated from 2019 to 2036 by setting the baseline year as the last year of the observed calendar year such that $\boldsymbol{\kappa}^*_1 = (\hat{\kappa}_{1,18}, \hat{\kappa}_{2,18})^T$ in (6).

Model validation is carried out both for LC and BC by quantifying Pearson residuals and checking with possible patterns across different ages and years for a given region and deprivation quintile, where appropriate. The residuals are obtained as follows:

$$r_{a,d,r,t} = \frac{D_{a,d,r,t} - \hat{\mathbb{E}}(D_{a,d,r,t})}{\sqrt{\hat{\text{var}}(D_{a,d,r,t})}},$$

where $\hat{\mathbb{E}}(D_{a,d,r,t}) = \hat{\theta}_{a,d,r,t} P_{a,d,r,t}$ and

$\hat{\text{var}}(D_{a,d,r,t}) = \hat{\mathbb{E}}(D_{a,d,r,t}) \times (1 + \hat{\mathbb{E}}(D_{a,d,r,t})\ \exp(\sigma^2 - 1))$ [10]. The corresponding fitted mortality rate, $\hat{\theta}_{a,d,r,t}$, is derived using the mean of lognormal distribution as $\hat{\theta}_{a,d,r,t} = \exp(\mu_{a,d,r,t} + \sigma^2/2)$.

Female lung cancer mortality

We have established a model, where the location parameter of lognormal distribution in Eq 4 is defined as

$$\begin{aligned} \mu^{\text{lung}}_{a,d,r,t} = \beta_0 + \beta_{1,a} + \beta_{2,r} + \beta_{3,d} + \beta_4 \text{AAD}^{\text{morbidity}}_{r,d} + \beta_{5,d,a} + \beta_{6,r,a} + \kappa_{1,t} + \\ (\kappa_{2,t} + \beta_{7,r})\text{AAD}^{\text{morbidity}}_{r,d} + \beta_8 \text{NS}^{\text{women}}_{a,t-20}. \end{aligned} \tag{8}$$

Note that Eq 8 has been determined with respect to a forward variable selection procedure. Here, $\beta_{1,a}$ is the age coefficient for age group a with levels $a = 1, 2, \ldots, 8$, where a maps to $\{45-54, 55-59, 60-64, \ldots, 85-89\}$; $\kappa_{1,t}$ is the coefficient associated with period t with levels $t = 1, 2, \ldots, 18$, where t maps to $\{2001, 2002, \ldots, 2018\}$; $\beta_{2,r}$ is the region coefficient for region r with levels $r = 1, 2, \ldots, 9$, where r maps to {North East, North West, Yorkshire and the Humber, East Midlands, West Midlands, East, London, South East and South West}; $\beta_{3,d}$ is the deprivation coefficient for quintile d with levels $d = 1, 2, \ldots, 5$, respectively; $\kappa_{2,t}$ is the coefficient of interaction between

period effect and AAD component; $\beta_{5,d,a}$ is the coefficient of interaction between age-at-death and deprivation quintile; and $\beta_{6,r,a}$, for the interaction between age-at-death and region; $\beta_{7,r}$ is the coefficient of interaction between region effect and AAD component, and β_8 is the coefficient for the NS prevalence rates.

Male lung cancer mortality

We have constructed the male LC mortality model as follows:

$$\mu^{\text{lung}}_{a,d,r,t} = \beta_0 + \beta_{1,a} + \beta_{2,r} + \beta_{3,d} + \beta_4 \text{AAD}^{\text{morbidity}}_{r,d} + \beta_{5,d,a} + \kappa_{1,t} + \big(\kappa_{2,t} + \beta_{6,r}\big)\text{AAD}^{\text{morbidity}}_{r,d} + \beta_7 \text{NS}^{\text{men}}_{a,t-20}, \quad (9)$$

where the main difference between the models in Eq 8 and Eq 9 causes due to the additional interaction term between age and region in the former model.

We note that the LC models in Eq 8 and Eq 9, for women and men, respectively, have been used to establish future scenarios associated with cancer diagnosis. Specifically, we have introduced diagnosis delays with the aid of AAD component and estimated related increases in LC mortality accordingly.

We also note that several model specifications have been implemented before determining the final modelling structure(s). This is because different best fitted models can be identified by changing the description of null model in the variable selection process. The overall decision has been made with the aim of finding a compromise between model complexity, data fitting, and potential correlations across different variables.

Female breast cancer mortality

We focus on female BC mortality as there are few records regarding male BC. It is important to note that, in the existence of other variables, e.g. age and region, income deprivation is not found to be a significant risk factor to explain differences in BC mortality in England, e.g. see [5]. Hereby, BC mortality projection is considered on regional level. Furthermore, the AAD variable by the regions of England has not been found to be statistically important to explain BC mortality either. This can be attributed to the empirical evidence, suggesting more ‘equality’ in BC mortality as compared to a lifestyle-related cancer, e.g. LC mortality. This results with comparable AAD estimates across different regions of England. Despite the fact that income deprivation and AAD variables were not significant for modelling purposes, female NS prevalence rates have been found to be an important risk factor that contributes to explain BC mortality.

Following the variable selection procedure, we have come up with a much simpler projection model as opposed to LC models such that

$$\mu^{\text{breast}}_{a,r,t} = \beta_0 + \beta_{1,a} + \beta_{2,r} + \beta_3 \text{NS}^{\text{women}}_{a,t-20} + \kappa_{1,t}. \quad (10)$$

Here, $\beta_{1,a}$ is the age coefficient for age group a with levels $a = 1, 2, \ldots, 11$, where a maps to $\{35-39, 40-44, 45-49, \ldots, 85-89\}$; $\kappa_{1,t}$ is the coefficient for the period component for period t with levels $t = 1, 2, \ldots, 18$, where t maps to $\{2001, 2002, \ldots, 2018\}$; $\beta_{2,r}$ is the coefficient of the region component; β_3 is the smoking coefficient.

Short-term variations in cancer deaths

Short-term variations, STV, in a given cancer type during the pandemic years (2020–2022) are examined by age and region through a comparison of observed versus predicted death counts. The calculation for a given gender across the regions r of England can be shown as follows:

$$\text{STV}_r = \frac{\sum_{t=2020}^{2022} \sum_d \sum_a D_{a,d,r,t}}{\sum_{t=2020}^{2022} \sum_d \sum_a \hat{\mathbb{E}}(D_{a,d,r,t}^{\text{baseline}})},$$

where $\hat{\mathbb{E}}(D_{a,d,r,t}^{\text{baseline}})$ refers to the pre-pandemic estimates with no COVID-impact. See Assumption 2 under 'Measures of excess deaths' regarding the calculation of $\hat{\mathbb{E}}(D_{a,d,r,t}^{\text{baseline}})$.

Measures of excess deaths

We examine excess cancer deaths based on our modelling framework, following a similar approach to the ONS [11]. Specifically, we investigate the impact of delays in cancer diagnosis on cancer mortality using AAD covariate. Provided that AAD has not been found important to explain differences in BC mortality, this part of the study is only relevant to LC mortality.

'Excess deaths' for a given gender from a certain cancer at various age groups a in deprivation level d and region r in the projection years, $\text{ED}_{a,d,r,t}$, are considered by subtracting the estimated number of deaths in the baseline calculations from those in a specific scenario as follows:

$$\text{ED}_{a,d,r,t} = \hat{\mathbb{E}}(D_{a,d,r,t}^{\text{scenario}}) - \hat{\mathbb{E}}(D_{a,d,r,t}^{\text{baseline}}).$$

Meanwhile, 'excess cause-specific cancer mortality' in the projection years is obtained by dividing excess cause-specific cancer deaths by the corresponding mid-year population estimates. Thus, in order to calculate age-specific excess cancer mortality for a given gender in a given projection year t, $\text{EAM}_{a,t}$, we use

$$\text{EAM}_{a,t} = \frac{\hat{\mathbb{E}}(D_{a,t}^{\text{scenario}}) - \hat{\mathbb{E}}(D_{a,t}^{\text{baseline}})}{P_{a,t}},$$

with, for instance, $\hat{\mathbb{E}}(D_{a,t}^{\text{scenario}}) = \sum_d \sum_r \hat{\mathbb{E}}(D_{a,d,r,t}^{\text{scenario}})$. In a similar manner, region-specific excess cancer mortality in year t, $\text{ERM}_{r,t}$, is obtained as

$$\text{ERM}_{r,t} = \frac{\hat{\mathbb{E}}(D_{r,t}^{\text{scenario}}) - \hat{\mathbb{E}}(D_{r,t}^{\text{baseline}})}{P_{r,t}}.$$

Last, deprivation-specific cancer mortality for a given gender in year t, $\text{EDM}_{d,t}^{c}$, is calculated as

$$\text{EDM}_{d,t} = \frac{\hat{\mathbb{E}}(D_{d,t}^{\text{scenario}}) - \hat{\mathbb{E}}(D_{d,t}^{\text{baseline}})}{P_{d,t}}.$$

Assumption 1: cancer survival

Under each scenario associated with diagnosis delays, we have taken into account for net cancer survival to distribute an overall increase (1- to 6-month) in AAD over time. Any increase in AAD in a given year would lead to an increase in cause-specific cancer mortality under inspection in the same year. Hereby, the aim is to allow a gradual

increase in the related cancer mortality in the future years. Particularly, we assume that a serious health disruption could cause a bigger increase in AAD in the first year of the projection by gradually declining later on.

LC survival is reported to gradually decrease over time such that 40% of people with LC would survive from this disease for one year or more, 15% for 5 years or more, and 10% for 10 years or more [12]. Hereby, we assume that a 60% increase of a particular delay, e.g. 1-month, in the AAD variable would realise in the second year of the projection period, 2020. This would be followed by a 25% increase up to 5 years, 10% from 6 to 10 years, and 5% in the rest of the projection period.

Assumption 2: population estimates in future years

Estimating cause-specific deaths in a particular future year requires to know both relevant mortality rates and mid-year population estimates. Our general modelling structure provides the framework to obtain mortality rates in the projection years (2019–2036). Nevertheless, for the calculation of the related number of deaths, the corresponding mid-year population estimates must be provided as well.

We rely on the national population projections provided by the ONS in the future years [13]. The population estimates, stratified by five-year age groups and gender in the regions of England, are available from 2019 to 2043. However, to facilitate our calculations, we specifically require the mid-year future population estimates by deprivation quintiles in a given region. Consequently, we make an additional assumption. We accept that the distribution of population estimates across deprivation quintiles within a given region in the last observed calendar year, 2018, would remain unchanged throughout the projected years.

References

1. Hovanec J, Siemiatycki J, Conway DI, Olsson A, Stücker I, Guida F, et al. Lung cancer and socioeconomic status in a pooled analysis of case-control studies. PLoS ONE. 2018;13(2).

2. Archbold M, Davies B, Mais D. Deprivation and the impact on smoking prevalence, England and Wales: 2017 to 2021. Office for National Statistics; 2023. Available from: `https://www.ons.gov.uk/peoplepopulationandcommunity/healthandsocialcare/drugusealcoholandsmoking/bulletins/deprivationandtheimpactonsmokingprevalenceenglandandwales/2017to2021`.

3. NHS Digital. Health Survey for England; 2020. Available from: `https://digital.nhs.uk/data-and-information/publications/statistical/health-survey-for-england/2019`.

4. Luo Q, O'Connell D, Yu XQ, Kahn C, Caruana M, et al. Cancer incidence and mortality in Australia from 2020 to 2044 and an exploratory analysis of the potential effect of treatment delays during the COVID-19 pandemic: a statistical modelling study. Lancet Public Health. 2022;7:537–48.

5. Arık A, Dodd E, Cairns A, Streftaris G. Socioeconomic disparities in cancer incidence and mortality in England and the impact of age-at-diagnosis on cancer mortality. PLoS One. 2021;16(7).

6. Brouhns N, Denuit M, Vermunt J. A Poisson log-bilinear regression approach to the construction of projected life tables. Insurance: Mathematics and Economics. 2002;31:373–393.

7. Brown JR. Redistribution and insurance: Mandatory annuitization with mortality heterogenity. Journal of Risk and Insurance. 2003;70(1):17–41.

8. Redondo Loures C, Cairns AJG. Mortality in the US by Education Level. Annals of Actuarial Science. 2019;14(2):384–419.

9. Arık A, Dodd E, Streftaris G. Cancer morbidity trends and regional differences in England - A Bayesian analysis. PLoS One. 2020;15(5).

10. Wong JST, Forster JJ, Smith PWF. Bayesian mortality forecasting with overdispersion. Insurance: Mathematics and Economics. 2018;83:206–221.

11. Office for National Statistics. Estimating excess deaths in the UK, methodology changes: February 2024; 2024. Available from: https://www.ons.gov.uk/peoplepopulationandcommunity/healthandsocialcare/causesofdeath/articles/estimatingexcessdeathsintheukmethodologychanges/february2024.

12. Cancer Research UK. Survival for all stages of lung cancer; 2021. Available from: https://www.cancerresearchuk.org/about-cancer/lung-cancer/survival.

13. Nash A. Subnational population projections for England: 2018-based. Office for National Statistics; 2020.

S1 Table. Estimated coefficients for lung cancer mortality in women.

Covariate	Parameter	Mean	SD	%2.5	%97.5	Covariate	Parameter	Mean	SD	%2.5	%97.5
	β_0	-7.2760	0.0264	-7.3240	-7.2260		$\beta_{5,\text{deprivation}_2,\text{age}_7}$	0.0859	0.0142	0.0573	0.1146
	β_{1,age_1}	-2.4300	0.0339	-2.4960	-2.3670		$\beta_{5,\text{deprivation}_3,\text{age}_7}$	-0.1328	0.0239	-0.1826	-0.0895
	β_{1,age_2}	-1.0340	0.0132	-1.0590	-1.0080		$\beta_{5,\text{deprivation}_4,\text{age}_7}$	-0.0941	0.0185	-0.1306	-0.0564
	β_{1,age_3}	-0.4921	0.0121	-0.5149	-0.4687		$\beta_{5,\text{deprivation}_5,\text{age}_7}$	-0.0649	0.0165	-0.0955	-0.0302
	β_{1,age_4}	-0.0717	0.0132	-0.0973	-0.0458		$\beta_{5,\text{deprivation}_1,\text{age}_8}$	-0.0852	0.0145	-0.1137	-0.0549
	β_{1,age_5}	0.3056	0.0133	0.2810	0.3316		$\beta_{5,\text{deprivation}_2,\text{age}_8}$	-0.0311	0.0137	-0.0575	-0.0029
	β_{1,age_6}	1.1480	0.0253	1.1010	1.1970		$\beta_{5,\text{deprivation}_3,\text{age}_8}$	0.0351	0.0140	0.0089	0.0631
	β_{1,age_7}	1.2830	0.0257	1.2340	1.3330		$\beta_{5,\text{deprivation}_4,\text{age}_8}$	0.1340	0.0141	0.1071	0.1613
	β_{1,age_8}	1.2920	0.0265	1.2410	1.3430		$\beta_{5,\text{deprivation}_5,\text{age}_8}$	0.2390	0.0160	0.2081	0.2696
	$\beta_{2,\text{region}_1}$	-0.1256	0.0386	-0.1850	-0.0552		$\beta_{6,\text{region}_1,\text{age}_1}$	-0.1074	0.0322	-0.1710	-0.0481
	$\beta_{2,\text{region}_2}$	0.1451	0.0073	0.1310	0.1592		$\beta_{6,\text{region}_2,\text{age}_1}$	-0.0668	0.0279	-0.1201	-0.0084
	$\beta_{2,\text{region}_3}$	0.0152	0.0145	-0.0096	0.0437		$\beta_{6,\text{region}_3,\text{age}_1}$	-0.0325	0.0243	-0.0784	0.0160
	$\beta_{2,\text{region}_4}$	0.1079	0.0154	0.0780	0.1358		$\beta_{6,\text{region}_4,\text{age}_1}$	-0.0331	0.0206	-0.0747	0.0063
	$\beta_{2,\text{region}_5}$	0.0113	0.0153	-0.0175	0.0378		$\beta_{6,\text{region}_5,\text{age}_1}$	0.0299	0.0192	-0.0067	0.0664
	$\beta_{2,\text{region}_6}$	0.0305	0.0136	0.0020	0.0534		$\beta_{6,\text{region}_6,\text{age}_1}$	0.0750	0.0182	0.0382	0.1086
	$\beta_{2,\text{region}_7}$	-0.5302	0.0344	-0.5821	-0.4674		$\beta_{6,\text{region}_7,\text{age}_1}$	0.0618	0.0192	0.0264	0.0992
	$\beta_{2,\text{region}_8}$	0.1846	0.0228	0.1423	0.2207		$\beta_{6,\text{region}_8,\text{age}_1}$	0.0731	0.0239	0.0264	0.1169
	$\beta_{2,\text{region}_9}$	0.1611	0.0293	0.1088	0.2087		$\beta_{6,\text{region}_9,\text{age}_1}$	-0.0718	0.0229	-0.1184	-0.0293
	$\beta_{3,\text{deprivation}_1}$	1.8050	0.1168	1.5980	1.9890		$\beta_{6,\text{region}_1,\text{age}_2}$	-0.0535	0.0200	-0.0918	-0.0136
	$\beta_{3,\text{deprivation}_2}$	0.7603	0.0488	0.6740	0.8345		$\beta_{6,\text{region}_2,\text{age}_2}$	0.0328	0.0175	-0.0020	0.0657
	$\beta_{3,\text{deprivation}_3}$	-0.0806	0.0070	-0.0937	-0.0668		$\beta_{6,\text{region}_3,\text{age}_2}$	0.0255	0.0165	-0.0068	0.0573
	$\beta_{3,\text{deprivation}_4}$	-0.8448	0.0547	-0.9253	-0.7480		$\beta_{6,\text{region}_4,\text{age}_2}$	0.0339	0.0147	0.0049	0.0623
	$\beta_{3,\text{deprivation}_5}$	-1.6400	0.1067	-1.8030	-1.4500		$\beta_{6,\text{region}_5,\text{age}_2}$	0.0355	0.0146	0.0063	0.0641
	β_4	0.9437	0.0803	0.8022	1.0700		$\beta_{6,\text{region}_6,\text{age}_2}$	0.0102	0.0152	-0.0185	0.0399
	$\beta_{5,\text{deprivation}_1,\text{age}_1}$	0.1318	0.0167	0.1005	0.1634		$\beta_{6,\text{region}_7,\text{age}_2}$	-0.0127	0.0173	-0.0457	0.0228
	$\beta_{5,\text{deprivation}_2,\text{age}_1}$	0.1150	0.0150	0.0869	0.1455		$\beta_{6,\text{region}_8,\text{age}_2}$	-0.0956	0.0264	-0.1475	-0.0420
	$\beta_{5,\text{deprivation}_3,\text{age}_1}$	0.0810	0.0132	0.0551	0.1069		$\beta_{6,\text{region}_9,\text{age}_2}$	0.0341	0.0216	-0.0098	0.0775
	$\beta_{5,\text{deprivation}_4,\text{age}_1}$	0.0797	0.0118	0.0563	0.1028		$\beta_{6,\text{region}_1,\text{age}_3}$	0.0281	0.0194	-0.0122	0.0655
	$\beta_{5,\text{deprivation}_5,\text{age}_1}$	0.0109	0.0116	-0.0109	0.0347		$\beta_{6,\text{region}_2,\text{age}_3}$	0.0046	0.0174	-0.0298	0.0394
	$\beta_{5,\text{deprivation}_1,\text{age}_2}$	-0.0592	0.0111	-0.0810	-0.0365		$\beta_{6,\text{region}_3,\text{age}_3}$	-0.0002	0.0160	-0.0323	0.0312
	$\beta_{5,\text{deprivation}_2,\text{age}_2}$	-0.1391	0.0114	-0.1616	-0.1171		$\beta_{6,\text{region}_4,\text{age}_3}$	0.0144	0.0153	-0.0162	0.0441
	$\beta_{5,\text{deprivation}_3,\text{age}_2}$	-0.2201	0.0135	-0.2458	-0.1924		$\beta_{6,\text{region}_5,\text{age}_3}$	-0.0042	0.0164	-0.0378	0.0269
	$\beta_{5,\text{deprivation}_4,\text{age}_2}$	0.0635	0.0177	0.0296	0.0973		$\beta_{6,\text{region}_6,\text{age}_3}$	0.0186	0.0188	-0.0167	0.0575
	$\beta_{5,\text{deprivation}_5,\text{age}_2}$	0.0298	0.0159	-0.0020	0.0608		$\beta_{6,\text{region}_7,\text{age}_3}$	0.0476	0.0299	-0.0083	0.1060
	$\beta_{5,\text{deprivation}_1,\text{age}_3}$	0.0403	0.0140	0.0127	0.0674		$\beta_{6,\text{region}_8,\text{age}_3}$	0.0372	0.0281	-0.0215	0.0929
	$\beta_{5,\text{deprivation}_2,\text{age}_3}$	0.0371	0.0119	0.0135	0.0591		$\beta_{6,\text{region}_9,\text{age}_3}$	0.0095	0.0221	-0.0307	0.0539
	$\beta_{5,\text{deprivation}_3,\text{age}_3}$	0.0263	0.0118	0.0026	0.0499		$\beta_{6,\text{region}_1,\text{age}_4}$	0.0305	0.0200	-0.0092	0.0698
	$\beta_{5,\text{deprivation}_4,\text{age}_3}$	-0.0126	0.0115	-0.0353	0.0081		$\beta_{6,\text{region}_2,\text{age}_4}$	0.0261	0.0180	-0.0077	0.0588
	$\beta_{5,\text{deprivation}_5,\text{age}_3}$	-0.0684	0.0121	-0.0930	-0.0440		$\beta_{6,\text{region}_3,\text{age}_4}$	-0.0153	0.0169	-0.0481	0.0172
	$\beta_{5,\text{deprivation}_1,\text{age}_4}$	-0.1159	0.0135	-0.1423	-0.0889		$\beta_{6,\text{region}_4,\text{age}_4}$	-0.0403	0.0190	-0.0764	-0.0021
	$\beta_{5,\text{deprivation}_2,\text{age}_4}$	-0.0237	0.0199	-0.0624	0.0146		$\beta_{6,\text{region}_5,\text{age}_4}$	-0.0953	0.0221	-0.1360	-0.0514
	$\beta_{5,\text{deprivation}_3,\text{age}_4}$	0.0030	0.0180	-0.0340	0.0378		$\beta_{6,\text{region}_6,\text{age}_4}$	0.0052	0.0272	-0.0475	0.0546
	$\beta_{5,\text{deprivation}_4,\text{age}_4}$	0.0151	0.0147	-0.0141	0.0434		$\beta_{6,\text{region}_7,\text{age}_4}$	0.0301	0.0285	-0.0256	0.0828
	$\beta_{5,\text{deprivation}_5,\text{age}_4}$	-0.0107	0.0137	-0.0367	0.0181		$\beta_{6,\text{region}_8,\text{age}_4}$	-0.0267	0.0217	-0.0687	0.0176
	$\beta_{5,\text{deprivation}_1,\text{age}_5}$	-0.0143	0.0125	-0.0393	0.0098		$\beta_{6,\text{region}_9,\text{age}_4}$	0.0088	0.0192	-0.0282	0.0471
	$\beta_{5,\text{deprivation}_2,\text{age}_5}$	0.0087	0.0116	-0.0139	0.0308		$\beta_{6,\text{region}_1,\text{age}_5}$	-0.0006	0.0168	-0.0320	0.0316
	$\beta_{5,\text{deprivation}_3,\text{age}_5}$	0.0108	0.0124	-0.0123	0.0351		$\beta_{6,\text{region}_2,\text{age}_5}$	0.0109	0.0170	-0.0204	0.0443
	$\beta_{5,\text{deprivation}_4,\text{age}_5}$	0.0111	0.0139	-0.0155	0.0368		$\beta_{6,\text{region}_3,\text{age}_5}$	0.0088	0.0179	-0.0261	0.0433
	$\beta_{5,\text{deprivation}_5,\text{age}_5}$	-0.0388	0.0207	-0.0820	0.0012		$\beta_{6,\text{region}_4,\text{age}_5}$	-0.0365	0.0215	-0.0781	0.0056
	$\beta_{5,\text{deprivation}_1,\text{age}_6}$	-0.0537	0.0183	-0.0893	-0.0169		$\beta_{6,\text{region}_5,\text{age}_5}$	0.0282	0.0304	-0.0336	0.0866
	$\beta_{5,\text{deprivation}_2,\text{age}_6}$	-0.0714	0.0151	-0.1018	-0.0433		$\beta_{6,\text{region}_6,\text{age}_5}$	0.0347	0.0250	-0.0139	0.0845
	$\beta_{5,\text{deprivation}_3,\text{age}_6}$	-0.0209	0.0133	-0.0459	0.0046		$\beta_{6,\text{region}_7,\text{age}_5}$	0.0055	0.0211	-0.0382	0.0485
	$\beta_{5,\text{deprivation}_4,\text{age}_6}$	0.0082	0.0130	-0.0176	0.0325		$\beta_{6,\text{region}_8,\text{age}_5}$	0.0028	0.0187	-0.0339	0.0393
	$\beta_{5,\text{deprivation}_5,\text{age}_6}$	0.0280	0.0125	0.0026	0.0522		$\beta_{6,\text{region}_9,\text{age}_5}$	-0.0058	0.0183	-0.0423	0.0295
	$\beta_{5,\text{deprivation}_1,\text{age}_7}$	0.0626	0.0122	0.0392	0.0874		$\beta_{6,\text{region}_1,\text{age}_6}$	-0.0362	0.0174	-0.0700	-0.0036

Covariate	Parameter	Mean	SD	%2.5	%97.5	Covariate	Parameter	Mean	SD	%2.5	%97.5
	$\beta_{6,\text{region}_2,\text{age}_6}$	-0.0016	0.0167	-0.0339	0.0316		$\kappa^*_{1,\text{year}_3}$	0.3224	0.0532	0.2238	0.4296
	$\beta_{6,\text{region}_3,\text{age}_6}$	-0.0274	0.0212	-0.0721	0.0120		$\kappa^*_{1,\text{year}_4}$	0.3353	0.0606	0.2211	0.4599
	$\beta_{6,\text{region}_4,\text{age}_6}$	-0.0904	0.0253	-0.1400	-0.0406		$\kappa^*_{1,\text{year}_5}$	0.3472	0.0655	0.2217	0.4758
	$\beta_{6,\text{region}_5,\text{age}_6}$	-0.1121	0.0240	-0.1575	-0.0633		$\kappa^*_{1,\text{year}_6}$	0.3596	0.0721	0.2189	0.5008
	$\beta_{6,\text{region}_6,\text{age}_6}$	-0.0584	0.0221	-0.1020	-0.0153		$\kappa^*_{1,\text{year}_7}$	0.3695	0.0796	0.2139	0.5235
	$\beta_{6,\text{region}_7,\text{age}_6}$	-0.0204	0.0191	-0.0569	0.0158		$\kappa^*_{1,\text{year}_8}$	0.3822	0.0854	0.2199	0.5491
	$\beta_{6,\text{region}_8,\text{age}_6}$	-0.0135	0.0167	-0.0482	0.0185		$\kappa^*_{1,\text{year}_9}$	0.3936	0.0915	0.2164	0.5855
	$\beta_{6,\text{region}_9,\text{age}_6}$	0.0310	0.0164	-0.0001	0.0641		$\kappa^*_{1,\text{year}_{10}}$	0.4060	0.0970	0.2287	0.6055
	$\beta_{6,\text{region}_1,\text{age}_7}$	0.1050	0.0175	0.0692	0.1369		$\kappa^*_{1,\text{year}_{11}}$	0.4174	0.1028	0.2307	0.6234
	$\beta_{6,\text{region}_2,\text{age}_7}$	0.1588	0.0204	0.1195	0.1991		$\kappa^*_{1,\text{year}_{12}}$	0.4292	0.1059	0.2389	0.6426
	$\beta_{6,\text{region}_3,\text{age}_7}$	0.0845	0.0238	0.0360	0.1307		$\kappa^*_{1,\text{year}_{13}}$	0.4405	0.1112	0.2390	0.6685
	$\beta_{6,\text{region}_4,\text{age}_7}$	0.0423	0.0221	0.0005	0.0867		$\kappa^*_{1,\text{year}_{14}}$	0.4509	0.1160	0.2306	0.6871
	$\beta_{6,\text{region}_5,\text{age}_7}$	0.0304	0.0188	-0.0089	0.0665		$\kappa^*_{1,\text{year}_{15}}$	0.4623	0.1203	0.2380	0.7114
	$\beta_{6,\text{region}_6,\text{age}_7}$	-0.0044	0.0165	-0.0358	0.0283		$\kappa^*_{1,\text{year}_{16}}$	0.4728	0.1240	0.2419	0.7243
	$\beta_{6,\text{region}_7,\text{age}_7}$	-0.0120	0.0162	-0.0433	0.0199		$\kappa^*_{1,\text{year}_{17}}$	0.4856	0.1271	0.2570	0.7562
	$\beta_{6,\text{region}_8,\text{age}_7}$	-0.0449	0.0157	-0.0749	-0.0154		$\kappa^*_{1,\text{year}_{18}}$	0.4956	0.1324	0.2544	0.7788
	$\beta_{6,\text{region}_9,\text{age}_7}$	-0.0635	0.0158	-0.0944	-0.0326		κ_{2,year_2}	-0.0178	0.0123	-0.0431	0.0049
	$\beta_{6,\text{region}_1,\text{age}_8}$	-0.0325	0.0175	-0.0665	0.0015		κ_{2,year_3}	-0.0189	0.0138	-0.0481	0.0090
	$\beta_{6,\text{region}_2,\text{age}_8}$	0.1998	0.0278	0.1482	0.2558		κ_{2,year_4}	-0.0251	0.0123	-0.0512	-0.0031
	$\beta_{6,\text{region}_3,\text{age}_8}$	0.0540	0.0254	0.0023	0.1022		κ_{2,year_5}	-0.0185	0.0129	-0.0425	0.0064
	$\beta_{6,\text{region}_4,\text{age}_8}$	0.0112	0.0220	-0.0292	0.0559		κ_{2,year_6}	-0.0268	0.0136	-0.0527	0.0010
	$\beta_{6,\text{region}_5,\text{age}_8}$	-0.0144	0.0193	-0.0520	0.0243		κ_{2,year_7}	-0.0200	0.0136	-0.0466	0.0057
	$\beta_{6,\text{region}_6,\text{age}_8}$	-0.0580	0.0181	-0.0923	-0.0219		κ_{2,year_8}	-0.0339	0.0136	-0.0593	-0.0076
	$\beta_{6,\text{region}_7,\text{age}_8}$	-0.0703	0.0182	-0.1059	-0.0338		κ_{2,year_9}	-0.0373	0.0143	-0.0672	-0.0100
	$\beta_{6,\text{region}_8,\text{age}_8}$	-0.0763	0.0191	-0.1114	-0.0373		$\kappa_{2,\text{year}_{10}}$	-0.0313	0.0138	-0.0589	-0.0062
	$\beta_{6,\text{region}_9,\text{age}_8}$	-0.0460	0.0210	-0.0862	-0.0023		$\kappa_{2,\text{year}_{11}}$	-0.0521	0.0134	-0.0801	-0.0268
	$\beta_{7,\text{region}_1}$	-0.0294	0.0098	-0.0480	-0.0103		$\kappa_{2,\text{year}_{12}}$	-0.0604	0.0131	-0.0861	-0.0358
	$\beta_{7,\text{region}_2}$	-0.0461	0.0068	-0.0598	-0.0326		$\kappa_{2,\text{year}_{13}}$	-0.0655	0.0141	-0.0946	-0.0384
	$\beta_{7,\text{region}_3}$	-0.0128	0.0070	-0.0267	0.0008		$\kappa_{2,\text{year}_{14}}$	-0.0664	0.0141	-0.0965	-0.0394
	$\beta_{7,\text{region}_4}$	0.0103	0.0085	-0.0065	0.0271		$\kappa_{2,\text{year}_{15}}$	-0.0774	0.0135	-0.1044	-0.0511
	$\beta_{7,\text{region}_5}$	0.0091	0.0076	-0.0058	0.0242		$\kappa_{2,\text{year}_{16}}$	-0.0811	0.0137	-0.1090	-0.0542
	$\beta_{7,\text{region}_6}$	0.0344	0.0085	0.0180	0.0518		$\kappa_{2,\text{year}_{17}}$	-0.0912	0.0140	-0.1199	-0.0643
	$\beta_{7,\text{region}_7}$	0.0741	0.0081	0.0584	0.0893		$\kappa_{2,\text{year}_{18}}$	-0.0975	0.0144	-0.1257	-0.0708
	$\beta_{7,\text{region}_8}$	-0.0145	0.0076	-0.0289	-0.0000		$\kappa^*_{2,\text{year}_1}$	-0.0997	0.0226	-0.1451	-0.0578
	$\beta_{7,\text{region}_9}$	-0.0252	0.0090	-0.0427	-0.0080		$\kappa^*_{2,\text{year}_2}$	-0.1022	0.0293	-0.1604	-0.0465
	β_8	-0.3542	0.0223	-0.3958	-0.3101		$\kappa^*_{2,\text{year}_3}$	-0.1053	0.0356	-0.1818	-0.0373
	κ_{1,year_2}	0.0235	0.0146	-0.0104	0.0471		$\kappa^*_{2,\text{year}_4}$	-0.1077	0.0411	-0.1975	-0.0262
	κ_{1,year_3}	0.0533	0.0162	0.0204	0.0830		$\kappa^*_{2,\text{year}_5}$	-0.1099	0.0459	-0.2049	-0.0220
	κ_{1,year_4}	0.0593	0.0159	0.0264	0.0871		$\kappa^*_{2,\text{year}_6}$	-0.1126	0.0510	-0.2145	-0.0076
	κ_{1,year_5}	0.1041	0.0163	0.0709	0.1347		$\kappa^*_{2,\text{year}_7}$	-0.1155	0.0550	-0.2309	-0.0057
	κ_{1,year_6}	0.1529	0.0147	0.1207	0.1791		$\kappa^*_{2,\text{year}_8}$	-0.1175	0.0590	-0.2329	0.0047
	κ_{1,year_7}	0.1924	0.0160	0.1578	0.2218		$\kappa^*_{2,\text{year}_9}$	-0.1198	0.0638	-0.2485	0.0106
	κ_{1,year_8}	0.2208	0.0165	0.1849	0.2482		$\kappa^*_{2,\text{year}_{10}}$	-0.1224	0.0678	-0.2559	0.0152
	κ_{1,year_9}	0.2292	0.0178	0.1940	0.2606		$\kappa^*_{2,\text{year}_{11}}$	-0.1263	0.0721	-0.2719	0.0126
	$\kappa_{1,\text{year}_{10}}$	0.2505	0.0181	0.2156	0.2848		$\kappa^*_{2,\text{year}_{12}}$	-0.1278	0.0760	-0.2891	0.0153
	$\kappa_{1,\text{year}_{11}}$	0.2641	0.0193	0.2258	0.3002		$\kappa^*_{2,\text{year}_{13}}$	-0.1312	0.0802	-0.2892	0.0197
	$\kappa_{1,\text{year}_{12}}$	0.2813	0.0200	0.2412	0.3181		$\kappa^*_{2,\text{year}_{14}}$	-0.1341	0.0845	-0.3026	0.0290
	$\kappa_{1,\text{year}_{13}}$	0.2906	0.0202	0.2498	0.3309		$\kappa^*_{2,\text{year}_{15}}$	-0.1363	0.0881	-0.3160	0.0328
	$\kappa_{1,\text{year}_{14}}$	0.3061	0.0208	0.2665	0.3448		$\kappa^*_{2,\text{year}_{16}}$	-0.1388	0.0923	-0.3199	0.0360
	$\kappa_{1,\text{year}_{15}}$	0.3145	0.0221	0.2719	0.3557		$\kappa^*_{2,\text{year}_{17}}$	-0.1407	0.0956	-0.3304	0.0452
	$\kappa_{1,\text{year}_{16}}$	0.3138	0.0234	0.2706	0.3566		$\kappa^*_{2,\text{year}_{18}}$	-0.1437	0.0998	-0.3472	0.0584
	$\kappa_{1,\text{year}_{17}}$	0.3049	0.0238	0.2587	0.3473		ψ_{κ_1}	0.0112	0.0040	0.0048	0.0204
	$\kappa_{1,\text{year}_{18}}$	0.2881	0.0240	0.2400	0.3329		ψ_{κ_2}	-0.0026	0.0032	-0.0095	0.0031
	$\kappa^*_{1,\text{year}_1}$	0.2999	0.0349	0.2328	0.3711		σ^2	0.0068	0.0007	0.0056	0.0082
	$\kappa^*_{1,\text{year}_2}$	0.3113	0.0455	0.2230	0.4041		$\sigma^2_{\psi_{\kappa_2}}$	0.0000	0.0000	0.0000	0.0000

S2 Table. Estimated coefficients for lung cancer mortality in men.

Covariate	Parameter	Mean	SD	%2.5	%97.5	Covariate	Parameter	Mean	SD	%2.5	%97.5
	β_0	-6.8200	0.0326	-6.8960	-6.7620		κ_{1,year_3}	0.0186	0.0105	-0.0019	0.0380
	β_{1,age_1}	-1.9120	0.0185	-1.9480	-1.8740		κ_{1,year_4}	0.0168	0.0119	-0.0041	0.0414
	β_{1,age_2}	-0.9831	0.0088	-1.0010	-0.9657		κ_{1,year_5}	0.0319	0.0122	0.0084	0.0568
	β_{1,age_3}	-0.3204	0.0074	-0.3352	-0.3056		κ_{1,year_6}	0.0637	0.0146	0.0364	0.0917
	β_{1,age_4}	-0.0378	0.0088	-0.0553	-0.0207		κ_{1,year_7}	0.0748	0.0142	0.0485	0.1053
	β_{1,age_5}	0.3721	0.0088	0.3539	0.3890		κ_{1,year_8}	0.1005	0.0164	0.0699	0.1361
	β_{1,age_6}	0.7774	0.0059	0.7657	0.7893		κ_{1,year_9}	0.1094	0.0168	0.0775	0.1448
	β_{1,age_7}	0.9769	0.0065	0.9640	0.9889		$\kappa_{1,\text{year}_{10}}$	0.1122	0.0198	0.0746	0.1508
	β_{1,age_8}	1.1270	0.0069	1.1140	1.1400		$\kappa_{1,\text{year}_{11}}$	0.1249	0.0196	0.0935	0.1699
	$\beta_{2,\text{region}_1}$	-0.5047	0.0110	-0.5255	-0.4826		$\kappa_{1,\text{year}_{12}}$	0.1298	0.0213	0.0920	0.1771
	$\beta_{2,\text{region}_2}$	0.1296	0.0052	0.1195	0.1400		$\kappa_{1,\text{year}_{13}}$	0.1469	0.0230	0.1062	0.1950
	$\beta_{2,\text{region}_3}$	-0.1027	0.0062	-0.1150	-0.0901		$\kappa_{1,\text{year}_{14}}$	0.1562	0.0252	0.1121	0.2073
	$\beta_{2,\text{region}_4}$	0.2865	0.0075	0.2714	0.3011		$\kappa_{1,\text{year}_{15}}$	0.1463	0.0263	0.0988	0.2029
	$\beta_{2,\text{region}_5}$	0.2578	0.0075	0.2427	0.2722		$\kappa_{1,\text{year}_{16}}$	0.1647	0.0285	0.1161	0.2254
	$\beta_{2,\text{region}_6}$	0.1515	0.0066	0.1383	0.1636		$\kappa_{1,\text{year}_{17}}$	0.1515	0.0295	0.0982	0.2175
	$\beta_{2,\text{region}_7}$	-0.8694	0.0108	-0.8904	-0.8468		$\kappa_{1,\text{year}_{18}}$	0.1456	0.0309	0.0915	0.2124
	$\beta_{2,\text{region}_8}$	0.3276	0.0070	0.3142	0.3420		$\kappa^*_{1,\text{year}_1}$	0.1509	0.0498	0.0554	0.2516
	$\beta_{2,\text{region}_9}$	0.3238	0.0084	0.3077	0.3407		$\kappa^*_{1,\text{year}_2}$	0.1573	0.0645	0.0349	0.2834
	$\beta_{3,\text{deprivation}_1}$	3.0050	0.0314	2.9370	3.0660		$\kappa^*_{1,\text{year}_3}$	0.1619	0.0760	0.0097	0.3099
	$\beta_{3,\text{deprivation}_2}$	1.2720	0.0134	1.2420	1.3000		$\kappa^*_{1,\text{year}_4}$	0.1665	0.0862	-0.0043	0.3388
	$\beta_{3,\text{deprivation}_3}$	-0.1210	0.0051	-0.1306	-0.1108		$\kappa^*_{1,\text{year}_5}$	0.1715	0.0960	-0.0191	0.3537
	$\beta_{3,\text{deprivation}_4}$	-1.4170	0.0151	-1.4450	-1.3850		$\kappa^*_{1,\text{year}_6}$	0.1760	0.1051	-0.0347	0.3808
	$\beta_{3,\text{deprivation}_5}$	-2.7390	0.0281	-2.7960	-2.6770		$\kappa^*_{1,\text{year}_7}$	0.1813	0.1155	-0.0516	0.4082
	β_4	1.7610	0.0214	1.7170	1.8040		$\kappa^*_{1,\text{year}_8}$	0.1860	0.1237	-0.0570	0.4335
	$\beta_{5,\text{deprivation}_1,\text{age}_1}$	0.1282	0.0135	0.1013	0.1543		$\kappa^*_{1,\text{year}_9}$	0.1911	0.1310	-0.0692	0.4480
	$\beta_{5,\text{deprivation}_2,\text{age}_1}$	0.1466	0.0127	0.1216	0.1720		$\kappa^*_{1,\text{year}_{10}}$	0.1961	0.1403	-0.0899	0.4747
	$\beta_{5,\text{deprivation}_3,\text{age}_1}$	0.1108	0.0105	0.0901	0.1320		$\kappa^*_{1,\text{year}_{11}}$	0.2001	0.1494	-0.1095	0.4897
	$\beta_{5,\text{deprivation}_4,\text{age}_1}$	0.0657	0.0090	0.0483	0.0828		$\kappa^*_{1,\text{year}_{12}}$	0.2075	0.1567	-0.1180	0.5154
	$\beta_{5,\text{deprivation}_5,\text{age}_1}$	-0.0059	0.0086	-0.0226	0.0115		$\kappa^*_{1,\text{year}_{13}}$	0.2133	0.1635	-0.1248	0.5335
	$\beta_{5,\text{deprivation}_1,\text{age}_2}$	-0.0708	0.0083	-0.0867	-0.0545		$\kappa^*_{1,\text{year}_{14}}$	0.2185	0.1692	-0.1257	0.5457
	$\beta_{5,\text{deprivation}_2,\text{age}_2}$	-0.1486	0.0093	-0.1670	-0.1302		$\kappa^*_{1,\text{year}_{15}}$	0.2232	0.1751	-0.1326	0.5643
	$\beta_{5,\text{deprivation}_3,\text{age}_2}$	-0.2260	0.0118	-0.2487	-0.2026		$\kappa^*_{1,\text{year}_{16}}$	0.2287	0.1814	-0.1341	0.5802
	$\beta_{5,\text{deprivation}_4,\text{age}_2}$	0.0638	0.0159	0.0316	0.0938		$\kappa^*_{1,\text{year}_{17}}$	0.2351	0.1881	-0.1330	0.5934
	$\beta_{5,\text{deprivation}_5,\text{age}_2}$	0.0445	0.0135	0.0164	0.0705		$\kappa^*_{1,\text{year}_{18}}$	0.2402	0.1940	-0.1444	0.6009
	$\beta_{5,\text{deprivation}_1,\text{age}_3}$	0.0278	0.0117	0.0051	0.0503		κ_{2,year_2}	0.0003	0.0138	-0.0270	0.0277
	$\beta_{5,\text{deprivation}_2,\text{age}_3}$	0.0391	0.0099	0.0191	0.0594		κ_{2,year_3}	0.0003	0.0137	-0.0256	0.0280
	$\beta_{5,\text{deprivation}_3,\text{age}_3}$	0.0052	0.0096	-0.0134	0.0240		κ_{2,year_4}	-0.0205	0.0126	-0.0448	0.0063
	$\beta_{5,\text{deprivation}_4,\text{age}_3}$	-0.0179	0.0091	-0.0359	-0.0008		κ_{2,year_5}	-0.0299	0.0119	-0.0521	-0.0057
	$\beta_{5,\text{deprivation}_5,\text{age}_3}$	-0.0710	0.0099	-0.0904	-0.0517		κ_{2,year_6}	-0.0348	0.0119	-0.0563	-0.0086
	$\beta_{5,\text{deprivation}_1,\text{age}_4}$	-0.0916	0.0121	-0.1157	-0.0680		κ_{2,year_7}	-0.0387	0.0140	-0.0686	-0.0129
	$\beta_{5,\text{deprivation}_2,\text{age}_4}$	0.0056	0.0176	-0.0276	0.0434		κ_{2,year_8}	-0.0480	0.0118	-0.0716	-0.0270
	$\beta_{5,\text{deprivation}_3,\text{age}_4}$	0.0044	0.0146	-0.0230	0.0339		κ_{2,year_9}	-0.0440	0.0123	-0.0695	-0.0218
	$\beta_{5,\text{deprivation}_4,\text{age}_4}$	-0.0242	0.0126	-0.0484	0.0015		$\kappa_{2,\text{year}_{10}}$	-0.0556	0.0118	-0.0773	-0.0290
	$\beta_{5,\text{deprivation}_5,\text{age}_4}$	0.0020	0.0100	-0.0178	0.0212		$\kappa_{2,\text{year}_{11}}$	-0.0511	0.0119	-0.0750	-0.0251
	$\beta_{5,\text{deprivation}_1,\text{age}_5}$	-0.0077	0.0098	-0.0275	0.0116		$\kappa_{2,\text{year}_{12}}$	-0.0532	0.0129	-0.0774	-0.0275
	$\beta_{5,\text{deprivation}_2,\text{age}_5}$	0.0100	0.0092	-0.0082	0.0279		$\kappa_{2,\text{year}_{13}}$	-0.0680	0.0125	-0.0938	-0.0437
	$\beta_{5,\text{deprivation}_3,\text{age}_5}$	0.0041	0.0096	-0.0150	0.0230		$\kappa_{2,\text{year}_{14}}$	-0.0798	0.0125	-0.1044	-0.0552
	$\beta_{5,\text{deprivation}_4,\text{age}_5}$	0.0057	0.0116	-0.0168	0.0295		$\kappa_{2,\text{year}_{15}}$	-0.0855	0.0123	-0.1082	-0.0605
	$\beta_{5,\text{deprivation}_5,\text{age}_5}$	-0.0834	0.0183	-0.1206	-0.0491		$\kappa_{2,\text{year}_{16}}$	-0.0804	0.0126	-0.1049	-0.0542
	$\beta_{5,\text{deprivation}_1,\text{age}_6}$	-0.0724	0.0165	-0.1048	-0.0397		$\kappa_{2,\text{year}_{17}}$	-0.0810	0.0122	-0.1039	-0.0552
	$\beta_{5,\text{deprivation}_2,\text{age}_6}$	-0.0474	0.0127	-0.0739	-0.0223		$\kappa_{2,\text{year}_{18}}$	-0.0847	0.0126	-0.1094	-0.0588
	$\beta_{5,\text{deprivation}_3,\text{age}_6}$	-0.0418	0.0103	-0.0617	-0.0216		$\kappa^*_{2,\text{year}_1}$	-0.0952	0.0395	-0.1749	-0.0175
	$\beta_{5,\text{deprivation}_4,\text{age}_6}$	0.0048	0.0096	-0.0143	0.0236		$\kappa^*_{2,\text{year}_2}$	-0.1065	0.0551	-0.2142	0.0033
	$\beta_{5,\text{deprivation}_5,\text{age}_6}$	0.0338	0.0095	0.0158	0.0525		$\kappa^*_{2,\text{year}_3}$	-0.1165	0.0656	-0.2447	0.0140
	$\beta_{5,\text{deprivation}_1,\text{age}_7}$	0.0784	0.0099	0.0592	0.0985		$\kappa^*_{2,\text{year}_4}$	-0.1271	0.0781	-0.2823	0.0249
	$\beta_{5,\text{deprivation}_2,\text{age}_7}$	0.1280	0.0129	0.1028	0.1531		$\kappa^*_{2,\text{year}_5}$	-0.1371	0.0882	-0.3114	0.0427
	$\beta_{5,\text{deprivation}_3,\text{age}_7}$	-0.1142	0.0209	-0.1592	-0.0742		$\kappa^*_{2,\text{year}_6}$	-0.1489	0.0966	-0.3392	0.0406
	$\beta_{5,\text{deprivation}_4,\text{age}_7}$	-0.1231	0.0189	-0.1605	-0.0868		$\kappa^*_{2,\text{year}_7}$	-0.1596	0.1049	-0.3703	0.0389
	$\beta_{5,\text{deprivation}_5,\text{age}_7}$	-0.0670	0.0145	-0.0934	-0.0366		$\kappa^*_{2,\text{year}_8}$	-0.1691	0.1131	-0.4038	0.0427
	$\beta_{5,\text{deprivation}_1,\text{age}_8}$	-0.0651	0.0120	-0.0878	-0.0422		$\kappa^*_{2,\text{year}_9}$	-0.1796	0.1216	-0.4359	0.0547
	$\beta_{5,\text{deprivation}_2,\text{age}_8}$	0.0036	0.0104	-0.0173	0.0228		$\kappa^*_{2,\text{year}_{10}}$	-0.1911	0.1281	-0.4500	0.0554
	$\beta_{5,\text{deprivation}_3,\text{age}_8}$	0.0448	0.0109	0.0238	0.0667		$\kappa^*_{2,\text{year}_{11}}$	-0.2015	0.1339	-0.4754	0.0511
	$\beta_{5,\text{deprivation}_4,\text{age}_8}$	0.1371	0.0114	0.1163	0.1594		$\kappa^*_{2,\text{year}_{12}}$	-0.2113	0.1400	-0.5031	0.0546
	$\beta_{5,\text{deprivation}_5,\text{age}_8}$	0.1839	0.0134	0.1570	0.2094		$\kappa^*_{2,\text{year}_{13}}$	-0.2212	0.1449	-0.5205	0.0590
	$\beta_{6,\text{region}_1}$	0.0010	0.0075	-0.0133	0.0164		$\kappa^*_{2,\text{year}_{14}}$	-0.2310	0.1517	-0.5447	0.0617
	$\beta_{6,\text{region}_2}$	-0.0040	0.0053	-0.0140	0.0061		$\kappa^*_{2,\text{year}_{15}}$	-0.2423	0.1571	-0.5715	0.0563
	$\beta_{6,\text{region}_3}$	0.0030	0.0058	-0.0085	0.0143		$\kappa^*_{2,\text{year}_{16}}$	-0.2542	0.1613	-0.5719	0.0402
	$\beta_{6,\text{region}_4}$	0.0201	0.0068	0.0069	0.0332		$\kappa^*_{2,\text{year}_{17}}$	-0.2656	0.1661	-0.6038	0.0482
	$\beta_{6,\text{region}_5}$	0.0074	0.0058	-0.0041	0.0190		$\kappa^*_{2,\text{year}_{18}}$	-0.2760	0.1699	-0.6196	0.0458
	$\beta_{6,\text{region}_6}$	0.0285	0.0063	0.0161	0.0406		σ^2	0.0020	0.0004	0.0014	0.0028
	$\beta_{6,\text{region}_7}$	0.0185	0.0062	0.0062	0.0309		$\sigma^2_{\psi_{\kappa_2}}$	0.0001	0.0000	0.0000	0.0002
	$\beta_{6,\text{region}_8}$	-0.0340	0.0058	-0.0454	-0.0228		$\sigma^2_{\psi_{\kappa_1}}$	0.0001	0.0000	0.0000	0.0002
	$\beta_{6,\text{region}_9}$	-0.0404	0.0066	-0.0533	-0.0275		$\sigma^2_{\kappa_1}$	0.0014	0.0005	0.0008	0.0028
	β_7	-0.3810	0.0207	-0.4265	-0.3425		$\sigma^2_{\kappa_2}$	0.0014	0.0005	0.0007	0.0027
	κ_{1,year_2}	0.0152	0.0101	-0.0037	0.0341						

S3 Table. Estimated coefficients for breast cancer mortality.

Covariate	Parameter	Mean	SD	%2.5	%97.5	Covariate	Parameter	Mean	SD	%2.5	%97.5
	β_0	-7.4490	0.0231	-7.4920	-7.3980		$\kappa_{1,\text{year}_{10}}$	-0.1507	0.0171	-0.1860	-0.1184
	β_{1,age_1}	-1.8890	0.0184	-1.9250	-1.8530		$\kappa_{1,\text{year}_{11}}$	-0.1618	0.0178	-0.1985	-0.1304
	β_{1,age_2}	-1.2540	0.0141	-1.2810	-1.2260		$\kappa_{1,\text{year}_{12}}$	-0.1756	0.0182	-0.2114	-0.1395
	β_{1,age_3}	-0.8577	0.0234	-0.9029	-0.8071		$\kappa_{1,\text{year}_{13}}$	-0.1973	0.0189	-0.2351	-0.1589
	β_{1,age_4}	-0.4689	0.0230	-0.5098	-0.4189		$\kappa_{1,\text{year}_{14}}$	-0.2132	0.0194	-0.2525	-0.1760
	β_{1,age_5}	-0.1101	0.0100	-0.1297	-0.0896		$\kappa_{1,\text{year}_{15}}$	-0.2164	0.0202	-0.2582	-0.1770
	β_{1,age_6}	0.0867	0.0098	0.0679	0.1056		$\kappa_{1,\text{year}_{16}}$	-0.2238	0.0206	-0.2666	-0.1841
	β_{1,age_7}	0.2495	0.0102	0.2295	0.2691		$\kappa_{1,\text{year}_{17}}$	-0.2360	0.0211	-0.2807	-0.1962
	β_{1,age_8}	0.4826	0.0104	0.4631	0.5027		$\kappa_{1,\text{year}_{18}}$	-0.2448	0.0225	-0.2911	-0.2019
	β_{1,age_9}	0.9480	0.0244	0.8951	0.9916		$\kappa^*_{1,\text{year}_1}$	-0.2530	0.0310	-0.3150	-0.1953
	$\beta_{1,\text{age}_{10}}$	1.2420	0.0240	1.1890	1.2840		$\kappa^*_{1,\text{year}_2}$	-0.2608	0.0373	-0.3341	-0.1875
	$\beta_{1,\text{age}_{11}}$	1.5710	0.0242	1.5170	1.6170		$\kappa^*_{1,\text{year}_3}$	-0.2688	0.0440	-0.3582	-0.1805
	$\beta_{2,\text{region}_1}$	-0.0316	0.0112	-0.0540	-0.0099		$\kappa^*_{1,\text{year}_4}$	-0.2758	0.0502	-0.3781	-0.1785
	$\beta_{2,\text{region}_2}$	-0.0144	0.0081	-0.0302	0.0013		$\kappa^*_{1,\text{year}_5}$	-0.2839	0.0558	-0.4041	-0.1789
	$\beta_{2,\text{region}_3}$	-0.0298	0.0091	-0.0479	-0.0117		$\kappa^*_{1,\text{year}_6}$	-0.2923	0.0597	-0.4121	-0.1759
	$\beta_{2,\text{region}_4}$	0.0234	0.0092	0.0053	0.0413		$\kappa^*_{1,\text{year}_7}$	-0.2997	0.0645	-0.4307	-0.1720
	$\beta_{2,\text{region}_5}$	0.0305	0.0084	0.0143	0.0470		$\kappa^*_{1,\text{year}_8}$	-0.3074	0.0693	-0.4450	-0.1677
	$\beta_{2,\text{region}_6}$	0.0283	0.0081	0.0123	0.0441		$\kappa^*_{1,\text{year}_9}$	-0.3154	0.0740	-0.4592	-0.1708
	$\beta_{2,\text{region}_7}$	-0.0101	0.0085	-0.0267	0.0066		$\kappa^*_{1,\text{year}_{10}}$	-0.3230	0.0776	-0.4727	-0.1717
	$\beta_{2,\text{region}_8}$	0.0189	0.0074	0.0045	0.0330		$\kappa^*_{1,\text{year}_{11}}$	-0.3302	0.0823	-0.4918	-0.1688
	$\beta_{2,\text{region}_9}$	-0.0152	0.0083	-0.0320	0.0006		$\kappa^*_{1,\text{year}_{12}}$	-0.3384	0.0870	-0.5060	-0.1723
	β_3	-0.1079	0.0181	-0.1409	-0.0673		$\kappa^*_{1,\text{year}_{13}}$	-0.3466	0.0910	-0.5243	-0.1722
	κ_{1,year_2}	-0.0137	0.0129	-0.0391	0.0131		$\kappa^*_{1,\text{year}_{14}}$	-0.3550	0.0949	-0.5470	-0.1732
	κ_{1,year_3}	-0.0396	0.0135	-0.0673	-0.0135		$\kappa^*_{1,\text{year}_{15}}$	-0.3631	0.0989	-0.5630	-0.1711
	κ_{1,year_4}	-0.0540	0.0146	-0.0835	-0.0265		$\kappa^*_{1,\text{year}_{16}}$	-0.3716	0.1029	-0.5788	-0.1732
	κ_{1,year_5}	-0.0585	0.0151	-0.0882	-0.0286		$\kappa^*_{1,\text{year}_{17}}$	-0.3793	0.1073	-0.6001	-0.1722
	κ_{1,year_6}	-0.0723	0.0150	-0.1019	-0.0426		$\kappa^*_{1,\text{year}_{18}}$	-0.3870	0.1116	-0.6129	-0.1692
	κ_{1,year_7}	-0.0954	0.0156	-0.1256	-0.0632		ψ_{κ_1}	-0.0081	0.0037	-0.0156	-0.0013
	κ_{1,year_8}	-0.1065	0.0159	-0.1373	-0.0743		σ^2	0.0038	0.0004	0.0031	0.0046
	κ_{1,year_9}	-0.1269	0.0158	-0.1578	-0.0961		$\sigma^2_{\kappa_1}$	0.0004	0.0002	0.0002	0.0009

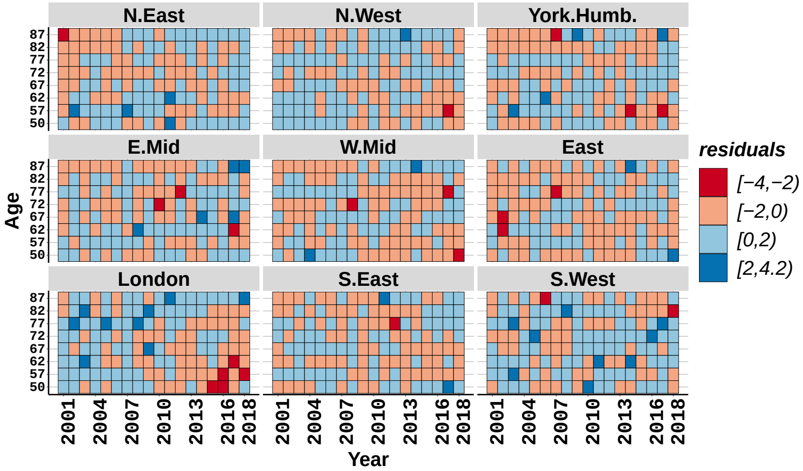

S5 Fig. Heat map of Pearson residuals for female lung cancer mortality in regions of England, deprivation quintile 1 (most deprived). Orange/light blue cells indicate areas with good fit, while red/dark blue cells indicate areas with poor fit. Note that there is a small number of residuals greater than 4, and these are included in the last category.

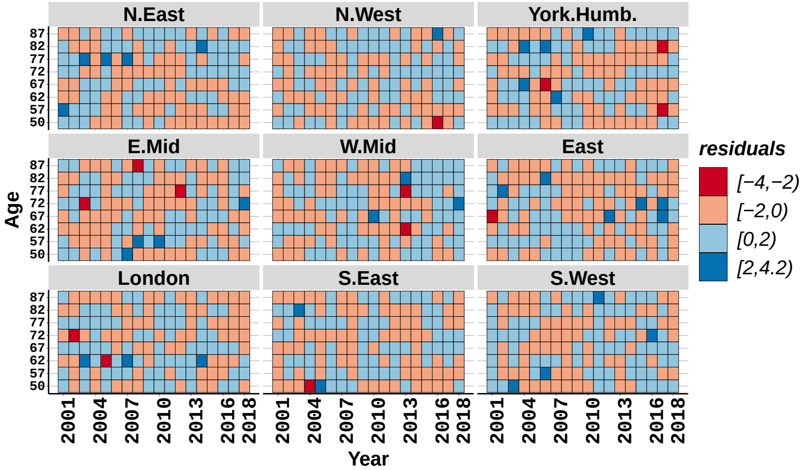

S6 Fig. Heat map of Pearson residuals for female lung cancer mortality in regions of England, deprivation quintile 5 (least deprived). Orange/light blue cells indicate areas with good fit, while red/dark blue cells indicate areas with poor fit. Note that there is a small number of residuals greater than 4, and these are included in the last category.

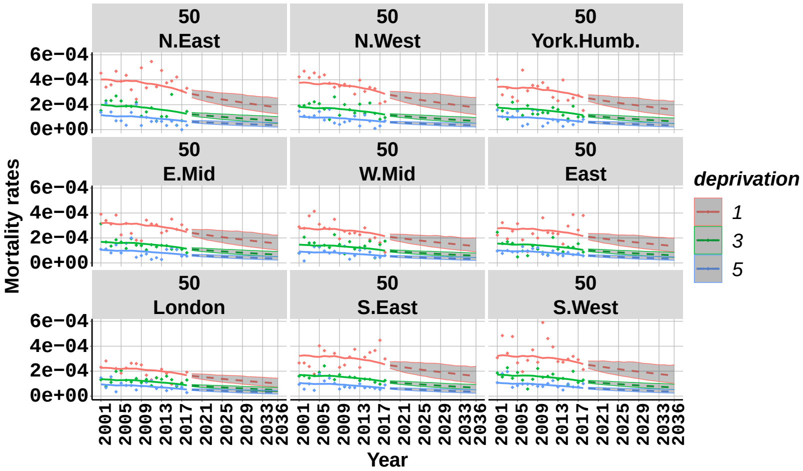

S7 Fig. Lung cancer mortality, females, ages at death 50, in selected deprivation quintiles 1 (most deprived), 3, and 5 (least deprived) in regions of England. Observed rates (dots), fitted rates (lines), projected rates (dashed lines) with 95% credible intervals for the projected rates.

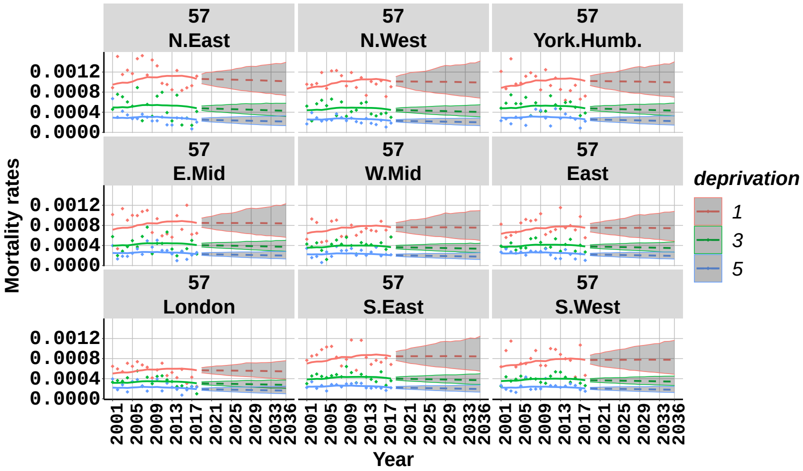

S8 Fig. Lung cancer mortality, females, ages at death 57, in selected deprivation quintiles 1 (most deprived), 3, and 5 (least deprived) in regions of England. Observed rates (dots), fitted rates (lines), projected rates (dashed lines) with 95% credible intervals for the projected rates.

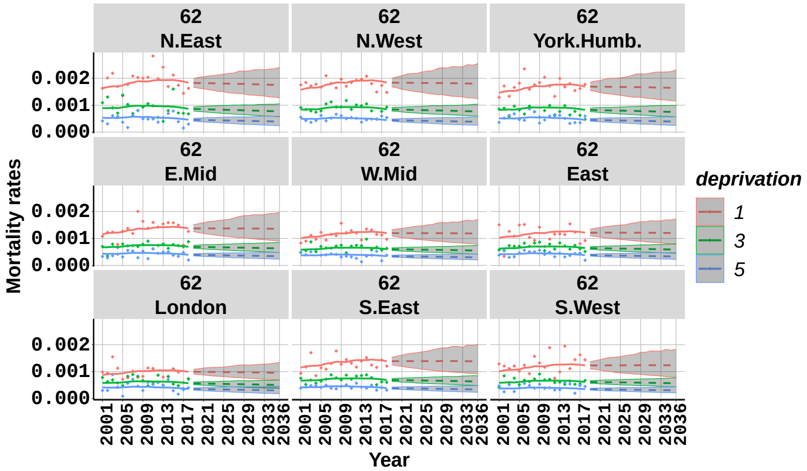

S9 Fig. Lung cancer mortality, females, ages at death 62, in selected deprivation quintiles 1 (most deprived), 3, and 5 (least deprived) in regions of England. Observed rates (dots), fitted rates (lines), projected rates (dashed lines) with 95% credible intervals for the projected rates.

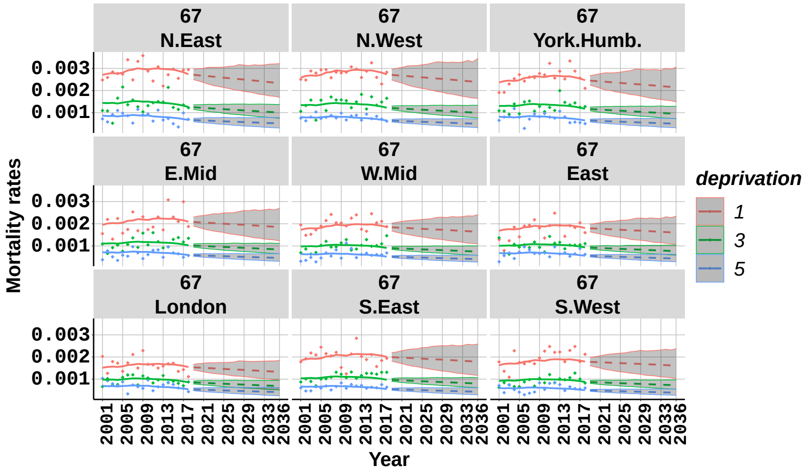

S10 Fig. Lung cancer mortality, females, ages at death 67, in selected deprivation quintiles 1 (most deprived), 3, and 5 (least deprived) in regions of England. Observed rates (dots), fitted rates (lines), projected rates (dashed lines) with 95% credible intervals for the projected rates.

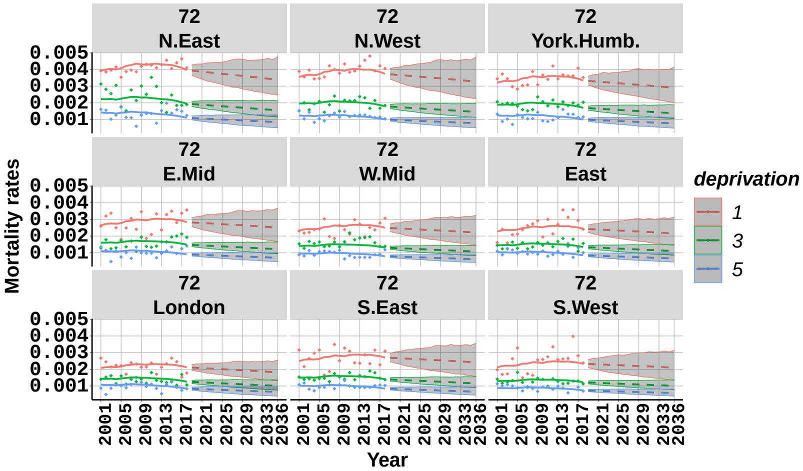

S11 Fig. Lung cancer mortality, females, ages at death 72, in selected deprivation quintiles 1 (most deprived), 3, and 5 (least deprived) in regions of England. Observed rates (dots), fitted rates (lines), projected rates (dashed lines) with 95% credible intervals for the projected rates.

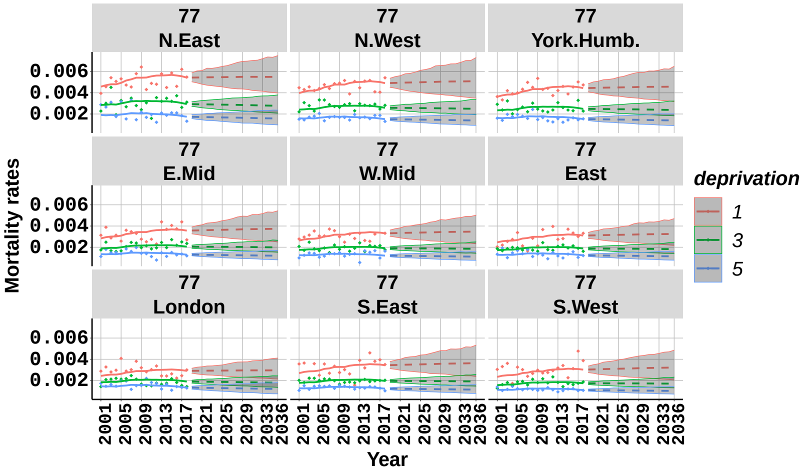

S12 Fig. Lung cancer mortality, females, ages at death 77, in selected deprivation quintiles 1 (most deprived), 3, and 5 (least deprived) in regions of England. Observed rates (dots), fitted rates (lines), projected rates (dashed lines) with 95% credible intervals for the projected rates.

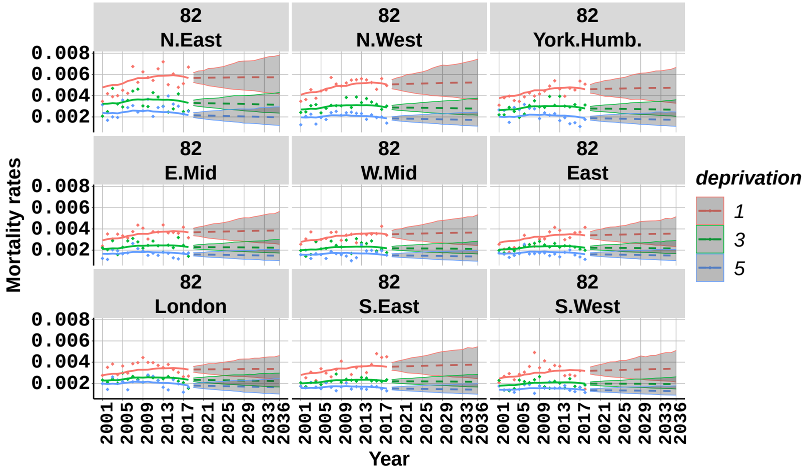

S13 Fig. Lung cancer mortality, females, ages at death 82, in selected deprivation quintiles 1 (most deprived), 3, and 5 (least deprived) in regions of England. Observed rates (dots), fitted rates (lines), projected rates (dashed lines) with 95% credible intervals for the projected rates.

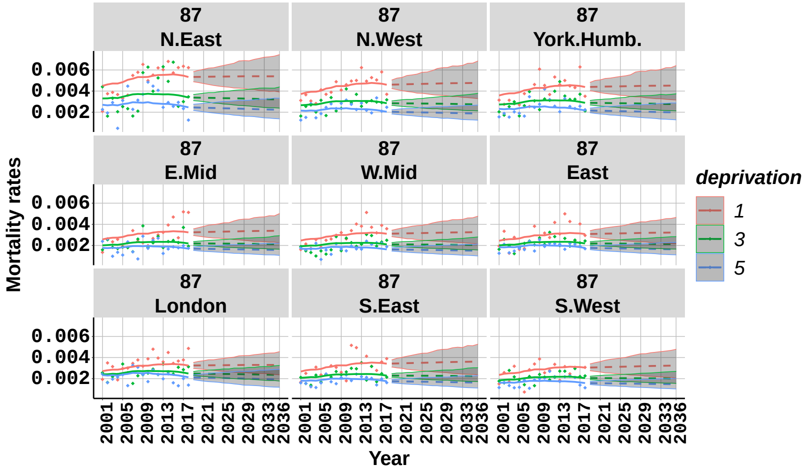

S14 Fig. Lung cancer mortality, females, ages at death 87, in selected deprivation quintiles 1 (most deprived), 3, and 5 (least deprived) in regions of England. Observed rates (dots), fitted rates (lines), projected rates (dashed lines) with 95% credible intervals for the projected rates.

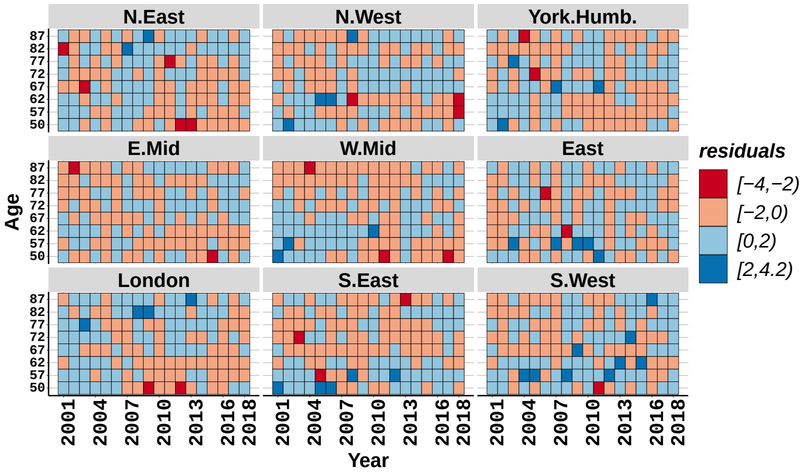

S15 Fig. Heat map of Pearson residuals for male lung cancer mortality in regions of England, deprivation quintile 1 (most deprived). Orange/light blue cells indicate areas with good fit, while red/dark blue cells indicate areas with poor fit. Note that there is a small number of residuals greater than 4, and these are included in the last category.

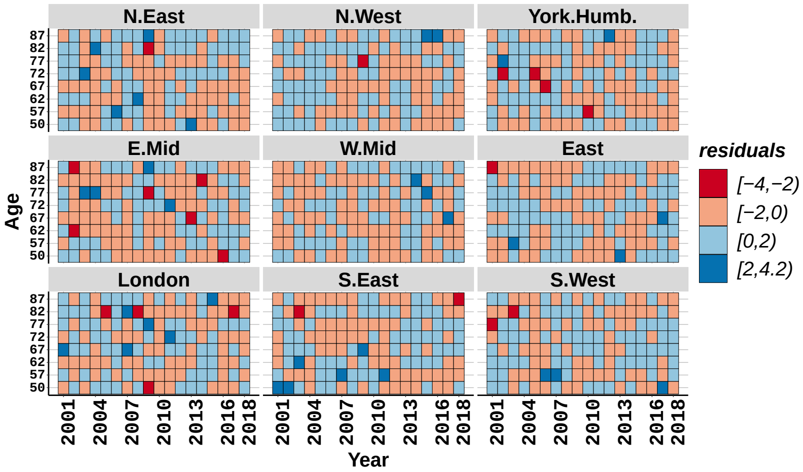

S16 Fig. Heat map of Pearson residuals for male lung cancer mortality in regions of England, deprivation quintile 5 (least deprived). Orange/light blue cells indicate areas with good fit, while red/dark blue cells indicate areas with poor fit. Note that there is a small number of residuals greater than 4, and these are included in the last category.

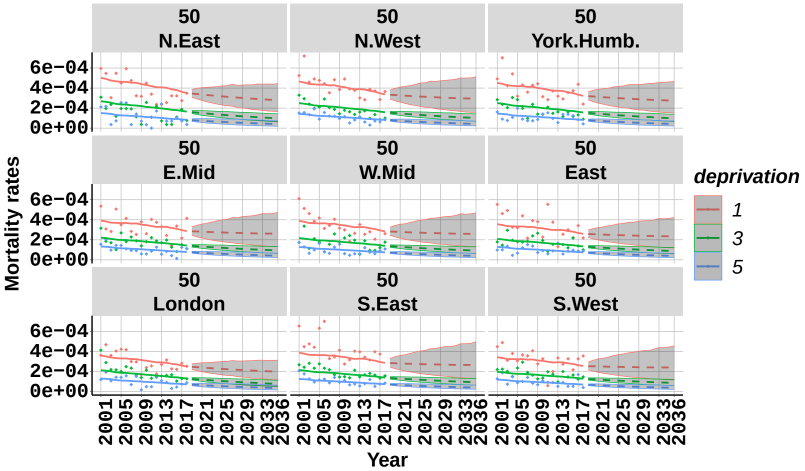

S17 Fig. Lung cancer mortality, males, ages at death 50, in selected deprivation quintiles 1 (most deprived), 3, and 5 (least deprived) in regions of England. Observed rates (dots), fitted rates (lines), projected rates (dashed lines) with 95% credible intervals for the projected rates.

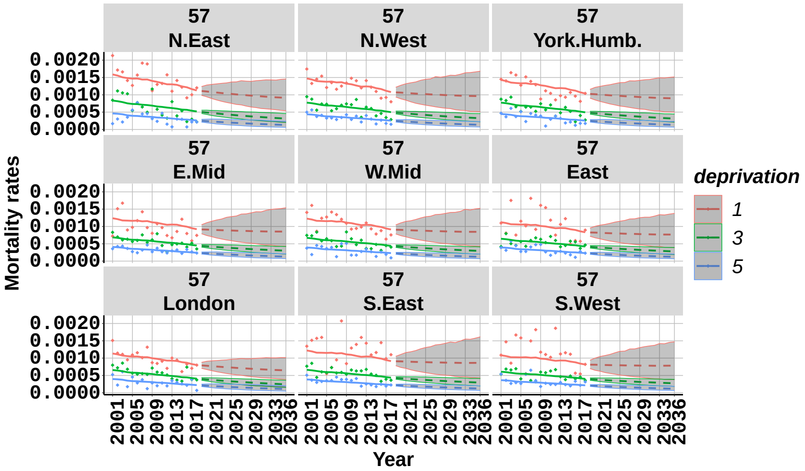

S18 Fig. Lung cancer mortality, males, ages at death 57, in selected deprivation quintiles 1 (most deprived), 3, and 5 (least deprived) in regions of England. Observed rates (dots), fitted rates (lines), projected rates (dashed lines) with 95% credible intervals for the projected rates.

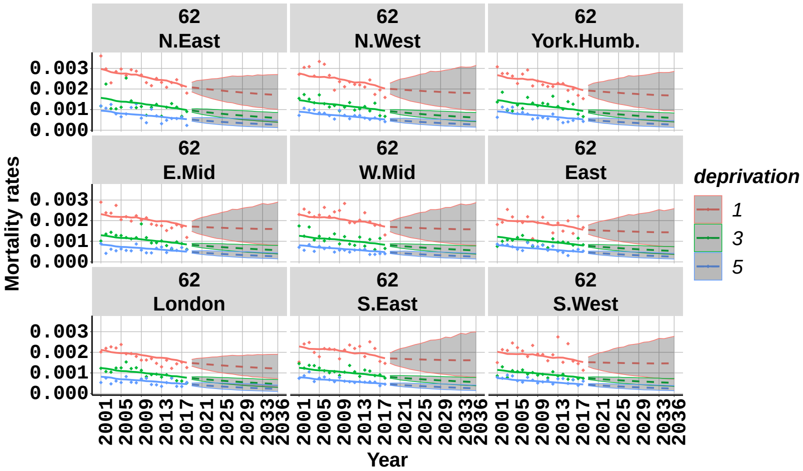

S19 Fig. Lung cancer mortality, males, ages at death 62, in selected deprivation quintiles 1 (most deprived), 3, and 5 (least deprived) in regions of England. Observed rates (dots), fitted rates (lines), projected rates (dashed lines) with 95% credible intervals for the projected rates.

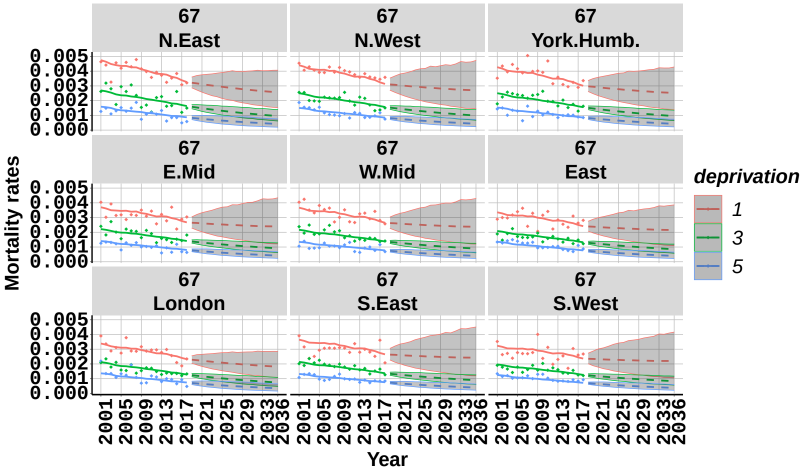

S20 Fig. Lung cancer mortality, males, ages at death 67, in selected deprivation quintiles 1 (most deprived), 3, and 5 (least deprived) in regions of England. Observed rates (dots), fitted rates (lines), projected rates (dashed lines) with 95% credible intervals for the projected rates.

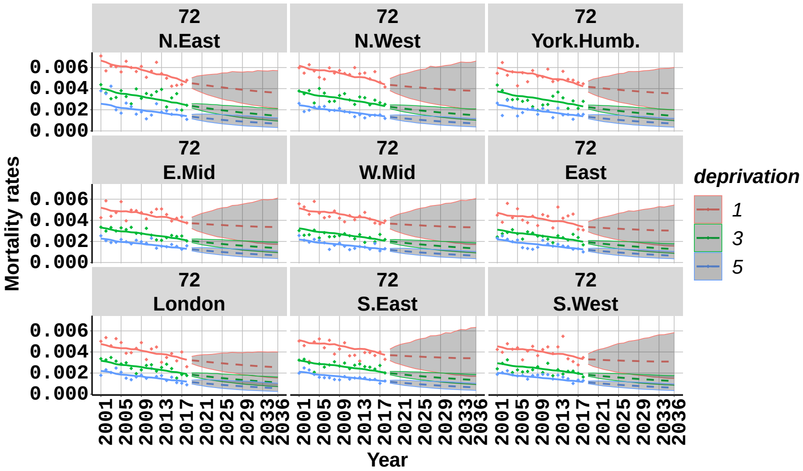

S21 Fig. Lung cancer mortality, males, ages at death 72, in selected deprivation quintiles 1 (most deprived), 3, and 5 (least deprived) in regions of England. Observed rates (dots), fitted rates (lines), projected rates (dashed lines) with 95% credible intervals for the projected rates.

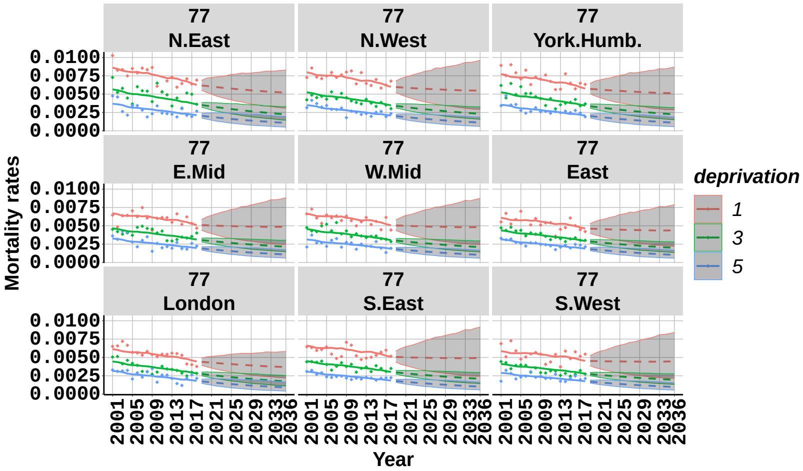

S22 Fig. Lung cancer mortality, males, ages at death 77, in selected deprivation quintiles 1 (most deprived), 3, and 5 (least deprived) in regions of England. Observed rates (dots), fitted rates (lines), projected rates (dashed lines) with 95% credible intervals for the projected rates.

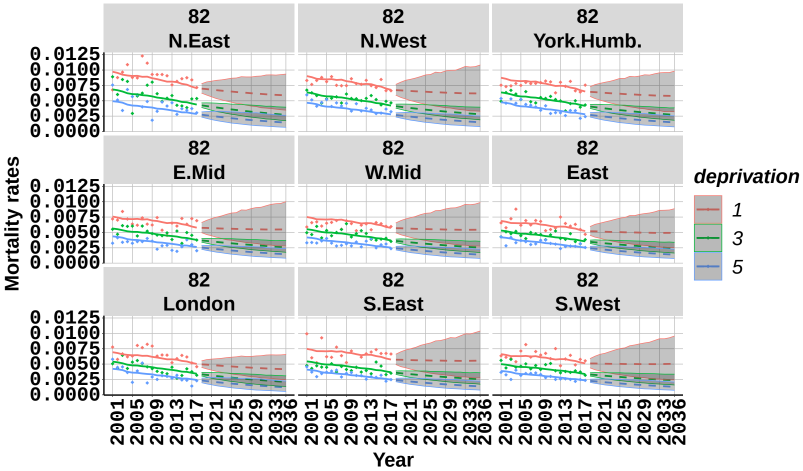

S23 Fig. Lung cancer mortality, males, ages at death 82, in selected deprivation quintiles 1 (most deprived), 3, and 5 (least deprived) in regions of England. Observed rates (dots), fitted rates (lines), projected rates (dashed lines) with 95% credible intervals for the projected rates.

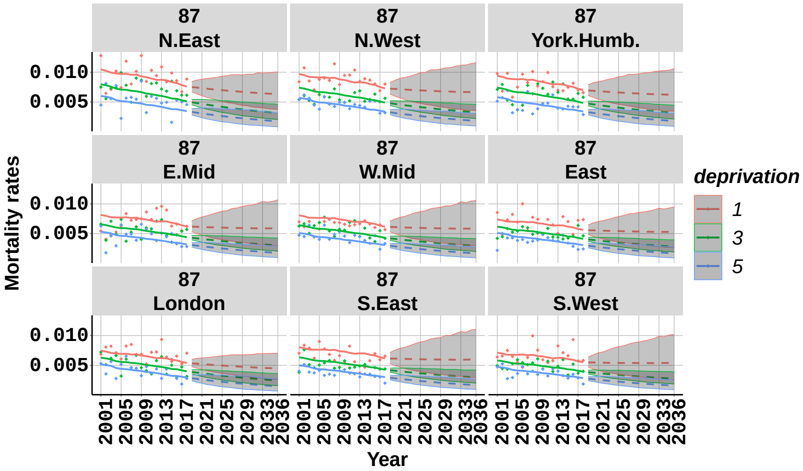

S24 Fig. Lung cancer mortality, males, ages at death 87, in selected deprivation quintiles 1 (most deprived), 3, and 5 (least deprived) in regions of England. Observed rates (dots), fitted rates (lines), projected rates (dashed lines) with 95% credible intervals for the projected rates.

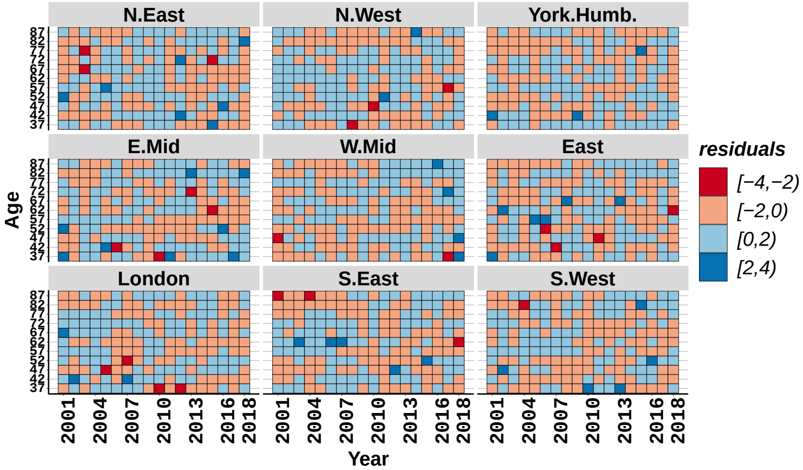

S25 Fig. Heat map of Pearson residuals for breast cancer mortality in regions of England. Orange/light blue cells indicate areas with good fit, while red/dark blue cells indicate areas with poor fit. Note that there is a small number of residuals greater than 4, and these are included in the last category.

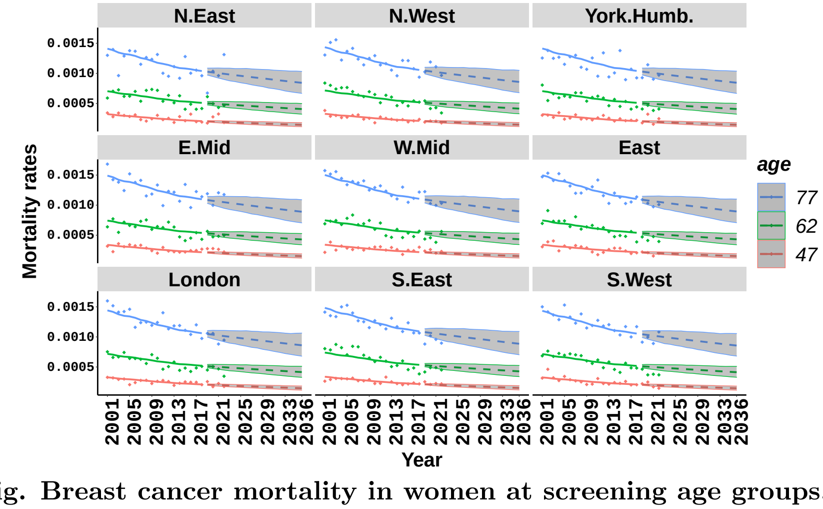

S26 Fig. Breast cancer mortality in women at screening age groups. Fitted (solid lines) and projected (dashed lines) rates, with 95% credible intervals, in regions of England.

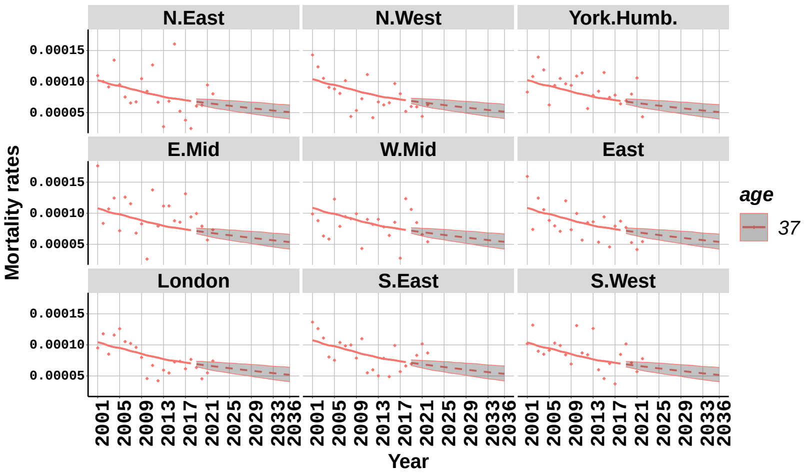

S27 Fig. Breast cancer mortality, females, age at death 37, in regions of England. Observed rates (dots), fitted rates (lines), projected rates (dashed lines) with 95% credible intervals for the projected rates.

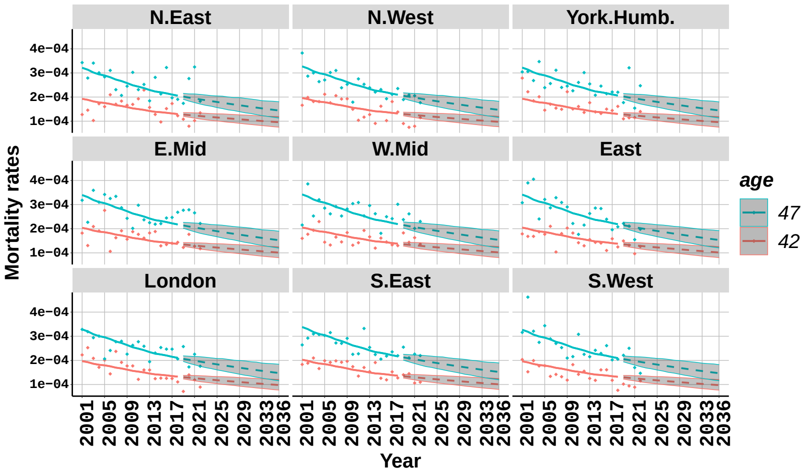

S28 Fig. Breast cancer mortality, females, ages at death 42 and 47, in regions of England. Observed rates (dots), fitted rates (lines), projected rates (dashed lines) with 95% credible intervals for the projected rates.

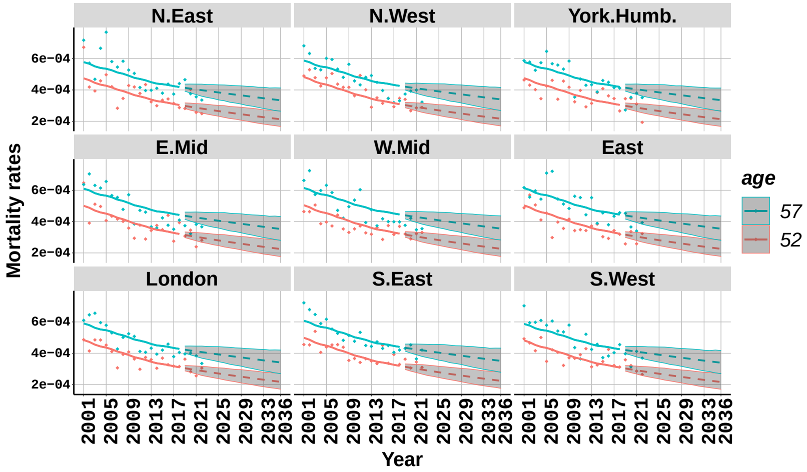

S29 Fig. Breast cancer mortality, females, ages at death 52 and 57, in regions of England. Observed rates (dots), fitted rates (lines), projected rates (dashed lines) with 95% credible intervals for the projected rates.

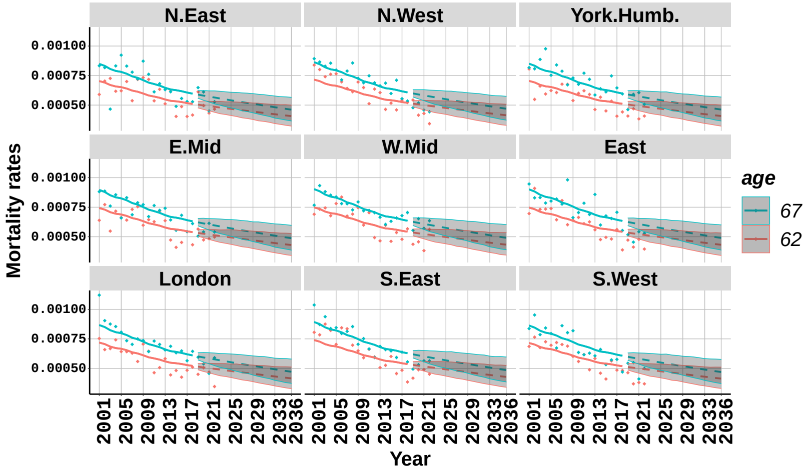

S30 Fig. Breast cancer mortality, females, ages at death 62 and 67, in regions of England. Observed rates (dots), fitted rates (lines), projected rates (dashed lines) with 95% credible intervals for the projected rates.

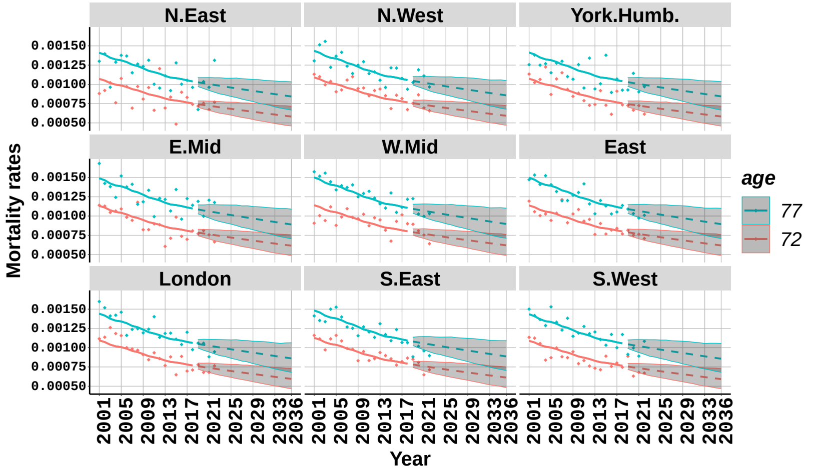

S31 Fig. Breast cancer mortality, females, ages at death 72 and 77, in regions of England. Observed rates (dots), fitted rates (lines), projected rates (dashed lines) with 95% credible intervals for the projected rates.

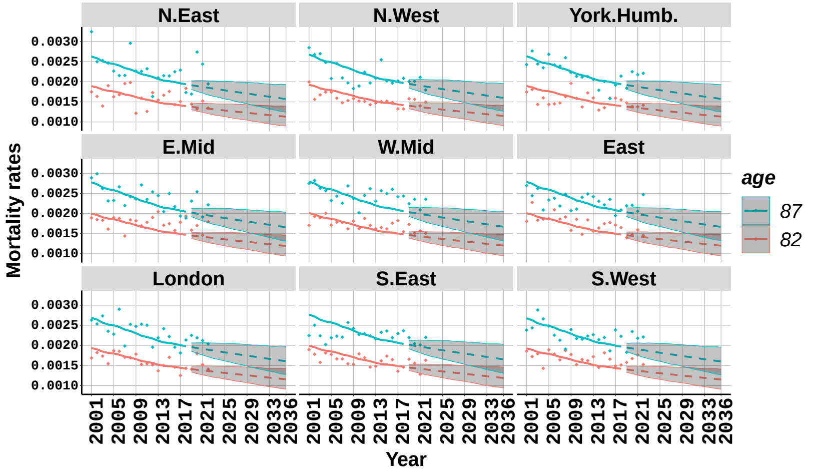

S32 Fig. Breast cancer mortality, females, ages at death 82 and 87, in regions of England. Observed rates (dots), fitted rates (lines), projected rates (dashed lines) with 95% credible intervals for the projected rates.

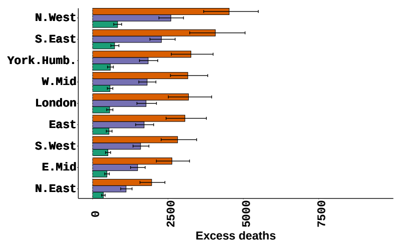

(a) Excess deaths, region

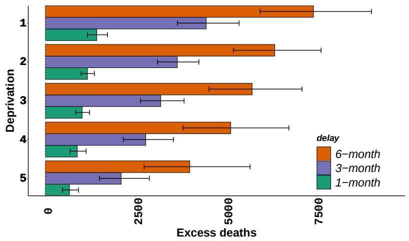

(b) Excess deaths, deprivation quintiles

S33 Fig. Cumulative lung cancer excess deaths for men from 2020 to 2036. Total excess deaths (over 17 years) in different deprivation quintiles ($\mathrm{ED}^{\mathrm{lung}}_{\mathrm{men},d}$) and regions of England ($\mathrm{ED}^{\mathrm{lung}}_{\mathrm{men},r}$), with 95% credible intervals.

S4 Table. Short-term variations in lung cancer deaths in men and women in the regions of England from 2020 to 2022, with 95% credible intervals.

	Female			Male		
	Registered deaths	Expected deaths	Ratio: registered/expected	Registered deaths	Expected deaths	Ratio: registered/expected
England	35250	36056.07	0.98 (0.89, 1.06)	39652	41664.99	0.96 (0.84, 1.09)
North East	2644	2889.65	0.92 (0.84, 1.00)	2663	2894.08	0.92 (0.81, 1.05)
North West	6119	6307.68	0.97 (0.88, 1.06)	6345	6734.76	0.95 (0.83, 1.08)
Yorkshire and the Humber	4349	4359.54	1.00 (0.91, 1.09)	4552	4773.26	0.96 (0.85, 1.09)
East Midlands	3270	3098.57	1.06 (0.96, 1.15)	3732	3755.71	1.00 (0.88, 1.14)
West Midlands	3702	3615.31	1.03 (0.93, 1.12)	4270	4648.58	0.92 (0.80, 1.06)
East of England	3564	3607.48	0.99 (0.90, 1.08)	4201	4331.23	0.97 (0.86, 1.10)
London	3057	3666.96	0.84 (0.76, 0.91)	3808	4317.56	0.89 (0.78, 1.00)
South East	5050	5122.42	0.99 (0.90, 1.08)	5893	6015.86	0.98 (0.87, 1.12)
South West	3495	3388.45	1.03 (0.93, 1.13)	4188	4193.96	1.00 (0.89, 1.14)

S5 Table. Short-term variations in lung cancer deaths at different ages in men and women England from 2020 to 2022, with 95% credible intervals.

	Female			Male		
	Registered deaths	Expected deaths	Ratio: registered/expected	Registered deaths	Expected deaths	Ratio: registered/expected
Ages 45–54	1239	1326.44	0.94 (0.84, 1.03)	1532	1632.44	0.94 (0.83, 1.08)
Ages 55–59	1782	2547.06	0.70 (0.63, 0.76)	2108	2602.80	0.81 (0.71, 0.93)
Ages 60–64	3195	3750.33	0.85 (0.77, 0.93)	3573	4263.46	0.84 (0.74, 0.96)
Ages 65–69	4588	4613.32	1.00 (0.90, 1.09)	5399	5622.19	0.96 (0.85, 1.10)
Ages 70–74	6946	6518.34	1.07 (0.97, 1.16)	7713	7848.12	0.99 (0.87, 1.12)
Ages 75–79	7318	7405.51	0.99 (0.90, 1.08)	8160	8632.90	0.95 (0.84, 1.08)
Ages 80–84	6183	5957.92	1.04 (0.95, 1.13)	6848	6681.51	1.03 (0.91, 1.17)
Ages 85–89	3999	3937.16	1.02 (0.92, 1.11)	4319	4381.57	0.99 (0.88, 1.13)

S6 Table. Short-term variations in lung cancer deaths in men and women in the regions of England from 2020 to 2022, with 90% credible intervals.

	Female			Male		
	Registered deaths	Expected deaths	Ratio: registered/expected	Registered deaths	Expected deaths	Ratio: registered/expected
England	35250	36056.07	0.98 (0.91, 1.05)	39652	41664.99	0.96 (0.86, 1.06)
North East	2644	2889.65	0.92 (0.85, 0.99)	2663	2894.08	0.92 (0.83, 1.03)
North West	6119	6307.68	0.97 (0.90, 1.05)	6345	6734.76	0.95 (0.85, 1.05)
Yorkshire and the Humber	4349	4359.54	1.00 (0.92, 1.07)	4552	4773.26	0.96 (0.86, 1.06)
East Midlands	3270	3098.57	1.06 (0.98, 1.14)	3732	3755.71	1.00 (0.90, 1.11)
West Midlands	3702	3615.31	1.03 (0.95, 1.11)	4270	4648.58	0.92 (0.82, 1.03)
East of England	3564	3607.48	0.99 (0.92, 1.06)	4201	4331.23	0.97 (0.88, 1.08)
London	3057	3666.96	0.84 (0.77, 0.90)	3808	4317.56	0.89 (0.80, 0.98)
South East	5050	5122.42	0.99 (0.91, 1.06)	5893	6015.86	0.98 (0.89, 1.09)
South West	3495	3388.45	1.03 (0.96, 1.11)	4188	4193.96	1.00 (0.90, 1.11)

S7 Table. Short-term variations in lung cancer deaths at different ages in men and women England from 2020 to 2022, with 90% credible intervals.

	Female			Male		
	Registered deaths	Expected deaths	Ratio: registered/expected	Registered deaths	Expected deaths	Ratio: registered/expected
Ages 45–54	1239	1326.44	0.94 (0.86, 1.02)	1532	1632.44	0.94 (0.84, 1.05)
Ages 55–59	1782	2547.06	0.70 (0.65, 0.75)	2108	2602.80	0.81 (0.73, 0.91)
Ages 60–64	3195	3750.33	0.85 (0.79, 0.92)	3573	4263.46	0.84 (0.76, 0.94)
Ages 65–69	4588	4613.32	1.00 (0.92, 1.07)	5399	5622.19	0.96 (0.87, 1.07)
Ages 70–74	6946	6518.34	1.07 (0.99, 1.15)	7713	7848.12	0.99 (0.89, 1.09)
Ages 75–79	7318	7405.51	0.99 (0.92, 1.06)	8160	8632.90	0.95 (0.86, 1.05)
Ages 80–84	6183	5957.92	1.04 (0.96, 1.12)	6848	6681.51	1.03 (0.93, 1.14)
Ages 85–89	3999	3937.16	1.02 (0.94, 1.09)	4319	4381.57	0.99 (0.89, 1.10)

S8 Table. Short-term variations in breast cancer deaths in the regions of England from 2020 to 2022, with 95% credible intervals.

	Registered deaths	Expected deaths	Ratio: registered/expected
England	24330	24640.51	0.99 (0.92, 1.06)
North East	1265	1206.33	1.05 (0.97, 1.13)
North West	3083	3207.73	0.96 (0.90, 1.04)
Yorkshire and the Humber	2365	2356.09	1.01 (0.93, 1.08)
East Midlands	2297	2243.39	1.03 (0.95, 1.11)
West Midlands	2694	2686.89	1.00 (0.93, 1.08)
East of England	2835	2967.60	0.96 (0.89, 1.03)
London	2795	2860.40	0.98 (0.91, 1.06)
South East	4262	4316.30	0.99 (0.92, 1.07)
South West	2734	2795.78	0.98 (0.91, 1.06)

S9 Table. Short-term variations in breast cancer deaths in the regions of England from 2020 to 2022, with 90% credible intervals.

	Registered deaths	Expected deaths	Ratio: registered/expected
England	24330	24640.51	0.99 (0.93, 1.05)
North East	1265	1206.33	1.05 (0.99, 1.12)
North West	3083	3207.73	0.96 (0.91, 1.02)
Yorkshire and the Humber	2365	2356.09	1.01 (0.95, 1.07)
East Midlands	2297	2243.39	1.03 (0.96, 1.09)
West Midlands	2694	2686.89	1.00 (0.94, 1.07)
East of England	2835	2967.60	0.96 (0.90, 1.02)
London	2795	2860.40	0.98 (0.92, 1.04)
South East	4262	4316.30	0.99 (0.93, 1.05)
South West	2734	2795.78	0.98 (0.92, 1.04)

S10 Table. Short-term variations in breast cancer deaths at different ages in England from 2020 to 2022, with 90% credible intervals.

	Registered deaths	Expected deaths	Ratio: registered/expected
Ages 35–39	391	395.05	0.99 (0.92, 1.06)
Ages 40–44	642	703.82	0.91 (0.86, 0.98)
Ages 45–49	1156	1094.09	1.06 (0.99, 1.13)
Ages 50–54	1755	1759.06	1.00 (0.93, 1.07)
Ages 55–59	2178	2427.11	0.90 (0.85, 0.96)
Ages 60–64	2192	2542.24	0.86 (0.81, 0.92)
Ages 65–69	2310	2547.13	0.91 (0.85, 0.96)
Ages 70–74	3099	3222.41	0.96 (0.91, 1.03)
Ages 75–79	3475	3533.54	0.98 (0.93, 1.05)
Ages 80–84	3673	3347.56	1.10 (1.03, 1.17)
Ages 85–89	3459	3068.50	1.13 (1.06, 1.20)

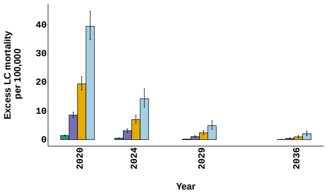

(a) 1-month delay, age-specific

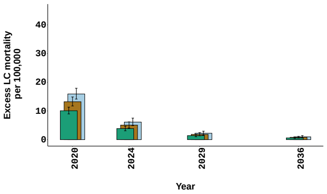

(b) 1-month delay, region-specific

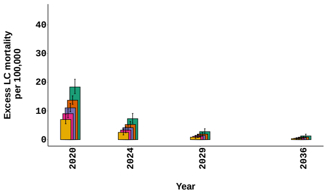

(c) 1-month delay, deprivation-specific

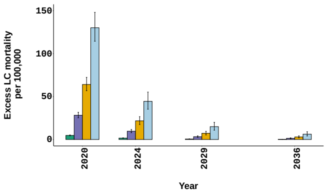

(d) 3-month delay, age-specific

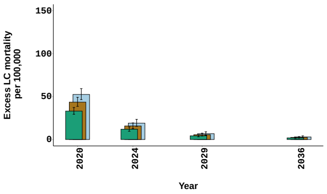

(e) 3-month delay, region-specific

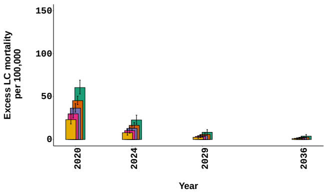

(f) 3-month delay, deprivation-specific

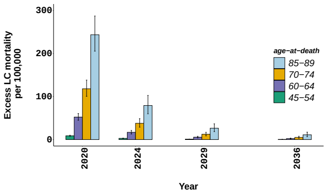

(g) 6-month delay, age-specific

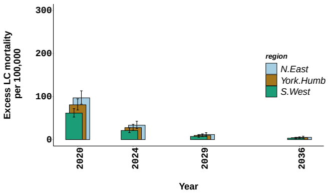

(h) 6-month delay, region-specific

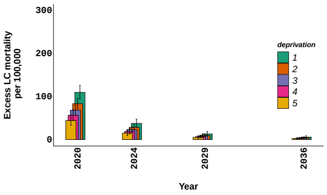

(i) 6-month delay, deprivation-specific

S34 Fig. Lung cancer excess mortality, per 100,000 men, by age-at-death ($\mathbf{EAM}^{\mathbf{lung}}_{a,\mathbf{men},t}$), selected regions ($\mathbf{ERM}^{\mathbf{lung}}_{\mathbf{men},r,t}$) and deprivation quintiles ($\mathbf{EDM}^{\mathbf{lung}}_{\mathbf{men},d,t}$) in England. Annual excess deaths from 2020 to 2036, with 95% credible intervals. Note that differences in lung cancer excess mortality at other ages, in other regions and deprivation quintiles in intermediate years, are comparable to the presented years.
